# Dynamic QTL-based ecophysiological models to predict phenotype from genotype and environment data

**DOI:** 10.1186/s12870-022-03624-7

**Published:** 2022-06-06

**Authors:** C. Eduardo Vallejos, James W. Jones, Mehul S. Bhakta, Salvador A. Gezan, Melanie J. Correll

**Affiliations:** 1 Horticultural Sciences Department, University of Florida, Gainesville, FL 32611 USA; 2 Plant Molecular and Cellular Biology Graduate Program, University of Florida, Gainesville, FL 32611 USA; 3 Department of Agricultural and Biological Engineering, University of Florida, Gainesville, FL 32611 USA; 4 School of Forest Resources and Conservation, University of Florida, Gainesville, FL 32611 USA; 5 Present Address: Bayer Crop Science, 700 Chesterfield Parkway, West Chesterfield, MO 63017 USA; 6 Present Address: VSN International, Hemel Hempstead, UK

**Keywords:** Common bean, *Phaseolus vulgaris*, Time-to-flowering, Mixed-effects model, Genotype-to-Phenotype prediction, Crop simulation models

## Abstract

**Background:**

Predicting the phenotype from the genotype is one of the major contemporary challenges in biology. This challenge is greater in plants because their development occurs mostly post-embryonically under diurnal and seasonal environmental fluctuations. Most current crop simulation models are physiology-based models capable of capturing environmental fluctuations but cannot adequately capture genotypic effects because they were not constructed within a genetics framework.

**Results:**

We describe the construction of a mixed-effects dynamic model to predict time-to-flowering in the common bean (*Phaseolus vulgaris* L.). This prediction model applies the developmental approach used by traditional crop simulation models, uses direct observational data, and captures the *Genotype*, *Environment*, and *Genotype-by-Environment* effects to predict progress towards time-to-flowering in real time. Comparisons to a traditional crop simulation model and to a previously developed static model shows the advantages of the new dynamic model.

**Conclusions:**

The dynamic model can be applied to other species and to different plant processes. These types of models can, in modular form, gradually replace plant processes in existing crop models as has been implemented in BeanGro, a crop simulation model within the DSSAT Cropping Systems Model. Gene-based dynamic models can accelerate precision breeding of diverse crop species, particularly with the prospects of climate change. Finally, a gene-based simulation model can assist policy decision makers in matters pertaining to prediction of food supplies.

**Supplementary Information:**

The online version contains supplementary material available at 10.1186/s12870-022-03624-7.

## Background

Gregor Mendel [1] deduced the genotype from the phenotype of garden peas. More recently, molecular characterization of Mendel’s genes [2] has underscored the feasibility of using the genotype to predict the phenotype—the G2P connection. However, prediction of complex phenotypes remains among the biggest challenges in biology today, and particularly in plants because they develop post-embryonically [3] under fluctuating environments resulting in different degrees of phenotypic plasticity [4]. Crop simulation models [5, 6] and quantitative trait locus (QTL) analysis [7–9] represent two complementary G2P approaches. While the former lacks a proper genetic framework, the latter doesn’t incorporate into the analysis the dynamic nature of the environment throughout the plant’s life cycle, despite existing dynamic methodologies [10]. These approaches have been combined previously with varying degrees of success [11–17].

The realization that model parameter values are genotype-specific led to the idea that they contain genetic information [11], which could be extracted from the parameters to turn them into mathematical functions of underlying genetic factors. Parameter estimation and optimization procedures produce values that confer high model predictability, but the resulting model may not entirely reflect the genetic architecture of the trait. For instance, it has been shown that different parameter sets within a chosen model structure may be nearly as good as a selected set of parameters in reproducing the observed behavior of a system [6, 18, 19].

However, model parameterization is based on some assumptions that may not be consistent with the genetic architecture of the associated biological process. For example, several models including APSIM [15] and CROPGRO [13] estimate developmental rates based primarily on thermal time as modified by photoperiod requirements, and vernalization in some cases. These parameters are based on daily mean temperatures and cardinal temperatures. The assumption that all genotypes of a species have the same cardinal temperatures contrasts with reports of genetic variation for cardinal temperatures [20–22]. Also, computation of mean daily temperatures overlooks the effect of daily thermocycling on biological processes that affect yield [23–25]. These observations suggest further testing of the hypothesis about the genetic information contained in model parameters.

Unlike traditional crop simulation models, statistical mixed-effects models offer an opportunity to predict phenotypic spectra using genotype (G), environmental (E), and GxE interactions data [26]. For example, Bhakta et al. [27] used only observational data to measure environmental and genetic effects to construct a static mixed-effects model for time-to-flowering (TTF) in the common bean. These effects were estimated in the absence of assumptions about the functional forms and parameters for the TTF trait. This model used 12 QTLs along with average maximum and minimum daily temperatures (Tmax and Tmin), average day length (Day), average daily solar radiation (SRAD), one QTLxQTL and seven specific QTLxE interactions.

**Fig. 1:**
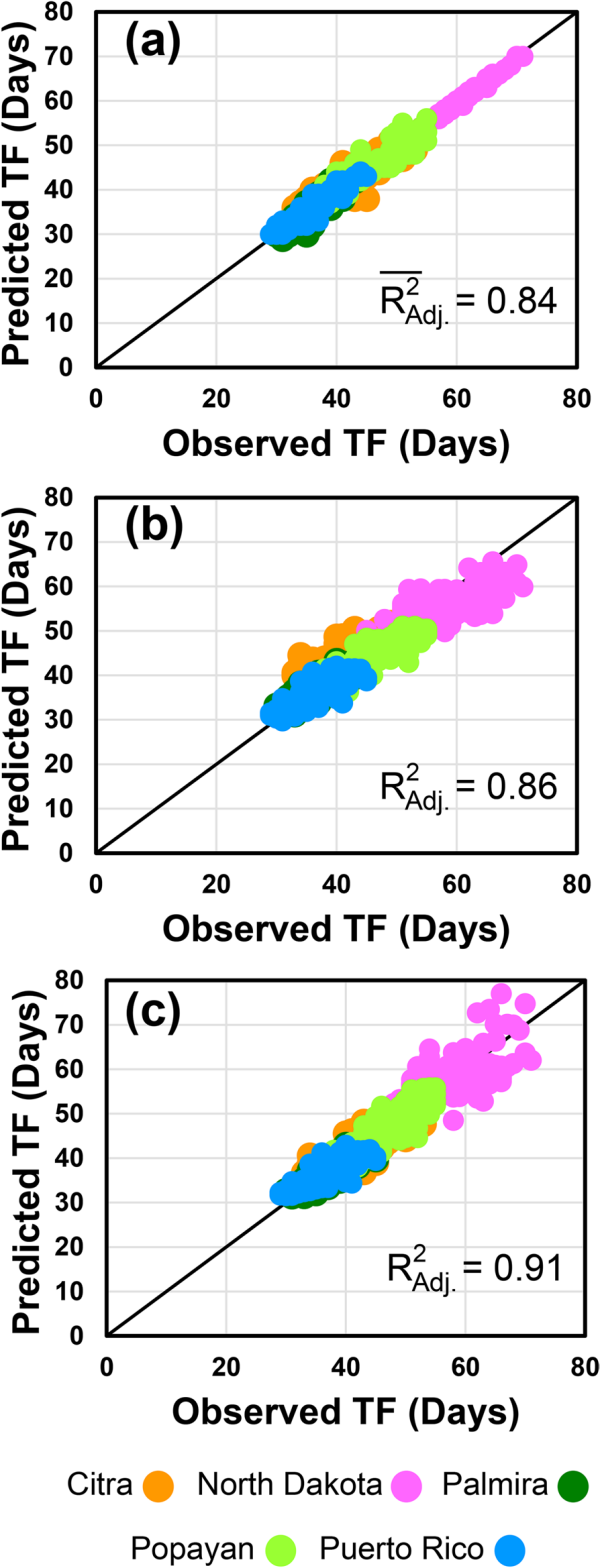
Observed vs. predicted time-to-flowering plots (1:1) of the experimental recombinant inbred family at the five sites. Predicted times were obtained with the CROPGRO-Bean model (**a**), the static (**b**) and the dynamic (**c**) statistical mixed-effects models. The R^2^_Adjusted_ value was used for comparative evaluation of the models to consider sample size and the number of model parameters. The the R^2^_Adjusted_ value for (a) is the average of the values calculated for each RIL that had observations at the five MET sites

The high predictability of this model (Fig. 1b) indicated that statistical models represent a data-driven modeling approach that provides a robust foundation for the development of gene-based crop simulation models. The estimated genetic effects could be divided into three categories. The first comprises environmentally stable genetic effects; the second includes the effects of specific QTLs on specific environmental responses, or GxE interactions, and the third includes the effect of one gene on the action of another gene on the phenotype, or epistatic interactions.

The mesothermic range of environments of the experimental sites allowed Bhakta et al. [27] to use linear functions for E effects. However, a more comprehensive effort will require a thorough survey of genotypic diversity through GWAS [28] and an analysis of the widest possible range of environments, which will likely demand the use of nonlinear functions [29]. This approach will also require the converging efforts of geneticists, statisticians, physiologists, and process-based dynamic crop modelers. It must be pointed out that the mixed-effects model approach is completely different from phenotypic prediction methodologies based on the genomic selection method developed by Meuwissen et al. [30], which uses all polymorphic markers for prediction purposes. We describe in this manuscript the conversion of a previously developed static mixed-effects model [27] into a dynamic model to predict the TTF phenotype in *Phaseolus vulgaris* using the same genotypic, phenotypic, and environmental data sets.

## Results

### Genetic analysis of crop model parameters

We selected the TTF trait to test the hypothesis that crop model parameters contain useful genetic information. We used the DSSAT CROPGRO-Bean model [31] to model the TTF trait of beans using data from a multi-environment trial (MET) carried out at five sites (Fargo, ND; Citra, FL; Isabela, PR; and Palmira and Popayan, Colombia. Table S1) with a recombinant inbred (RI) family (F_11:14_, n = 188) of *P. vulgaris*, L. [27, 32]. The TTF trait was selected because it has high heritability [27, 33]. More specifically, Bhakta et al. [27] reported that the overall broad sense heritability across five distinct environments was 0.786, while the site-specific heritabilities ranged from 0.690 to 0.890. This trait is also very responsive to environmental variables [34].

Like many crop models [13, 35–38], CROPGRO [31] handles daily environmental fluctuations using a development rate concept based on the following equation,

1 $$dP(t)/ dt=\left( 1/{DUR}_{min}\right)\ast f\left(T(t)\right)\ast g\left( DL(t), CSD, PPSEN\right)$$

where *dP*(*t*)/*dt* is the daily rate of progress toward flowering at time *t* (in days), *DUR*_*min*_ is the genotype-specific parameter (GSP) representing TTF under optimal temperature (T) and day length (DL) conditions during the entire time period, *f(T(t))* is a T-dependent function that reduces developmental progress rate on day *t*, and *g* is a DL-dependent function that modifies the progress rate on day *t. CSD* and *PPSEN* are GSPs that express genotype-specific sensitivity to DL; *CSD* is the genotype’s critical day length below which development is optimal, and *PPSEN* is the rate at which development rate is reduced for each hour above *CSD* (short-day plants). This rate is integrated over time (hours or days) until a developmental threshold is reached for simulating timing of a developmental transition such as TTF.

We applied inverse modeling [19] to estimate the three GSPs associated with TTF (PL-EM: planting-to-emergence; EM-FL: emergence-to-flowering; and PPSEN: photoperiod sensitivity) for each RI line (Table S2); MET phenotypes and meteorological data can be found in Additional file 1*.* CROPGRO used these GSPs and daily environmental data from MET sites as inputs to simulate TTF. The observed vs. simulated plot (Fig. 1a) appears to show that the model has high predictability, raising the possibility that flowering-associated GSPs may contain substantial genetic information.

QTL analyses of PL-EM, EM-FL and PPSEN in the RI family identified three, four and one QTL, respectively (Table S3). These QTLs only explained between 30 and 50% of all the observed variation (Table S3), and only four of them co-localized with one third of QTLs detected through QTL analysis of the TTF phenotypes reported by Bhakta et al*.* [27]. This observation indicated that not all the information in GSPs is genetically tractable.

### Development of a dynamic mixed-effects model

As an alternative to the use of the traditional crop simulation model for predicting the phenotype from the genotype under varying environmental conditions, we revisited the mixed-effects model developed by Bhakta et al. [27]. A limitation of that model is its static nature because it uses the average values of E factors recorded during trait development. This approach cannot adequately represent the dynamic nonlinear and time-varying environmental effects on plant processes. However, the static model can be transformed into a dynamic gene-based process simulation module by applying the developmental rate concept described by Eq. (1). An advantage of this application is that while TTF displays a curvilinear response to environmental factors, the rate of developmental progress exhibits a more linear response, at least under mesothermic conditions, resulting in an improved fit. This is basically the traditional crop modeling approach, which uses the duration of a developmental stage (TTF in days) to compute a rate of progress (RF(day^−1^) = 1/TTF) [31, 39]. A fundamental assumption in this approach is that the rate of progress depends only on the environmental conditions of each day, and that these dependencies remain constant during the entire phase that is being modeled. Dynamic models are commonly divided into developmental/phenological phases with developmental transitions timed by this modeling approach.

**Fig. 2:**
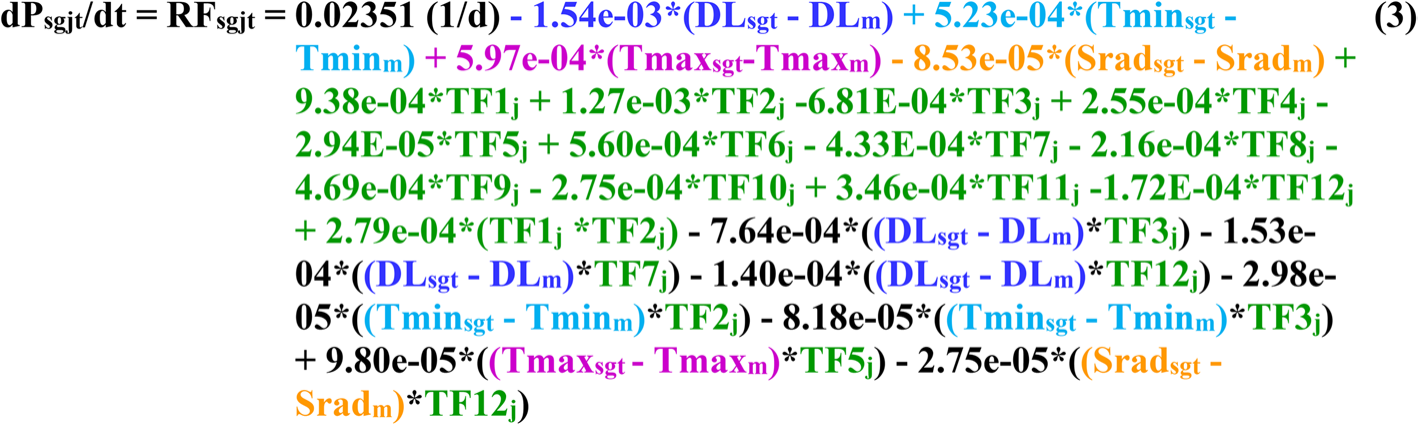
Differential equation describing the rate of development (RF) towards time-to-flowering as a function of genotype (g) and environmental (Tmax, Tmin, DL and Srad) factors at five experimental sites (s). This equation is represented by a linear mixed-effects function where: DL_sgt_ = day length (hours) experienced by genotype *g*^*th*^ on day *t* at site *s*, DL_m_ = mean day length across all five sites (12.37 h); Srad_sgt_ = solar radiation (Srad, MJ m^−2^ d^−1^) experienced by genotype *g*^*th*^ on day *t* at site *s*, Srad_m_ = mean solar radiation across all five sites (18.218 MJ m^−2^ d^−1^); Tmin_sgt_ = minimum temperature (°C) experienced by genotype *g*^*th*^ on day *t* at site *s*, Tmin_m_ = mean minimum temperature across all five sites (16.128 °C); Tmax_sgt_ = maximum temperature (°C) experienced by genotype *g*^*th*^ on day *t* at ste *s*, Tmax_m_ = mean maximum temperature across all five sites (27.458 °C); TF1_j_ to TF12_j_ = QTL operators for the *j*^*th*^ allele (Calima alleles = +1 and Jamapa alleles = −1). For the rate equation: 0.02351 (d^−1^) is the mean effect of the environmental factors. QTL parameters are in green, and parameters of each environmental factor have their own color

We constructed a mixed-effects linear model of development rate (RF) as a function of G, E, and GxE interactions (See Eq. (4) in the Methods section). This approach models the trait as a developmental rate, rather than the occurrence of an event at a time point. Accordingly, the overriding assumption is that the genetic factors in the model are controlling a dynamic trait. Fitting the generic model to data from the MET yielded a function with 25 parameters (Table 1; Fig. 2) describing the effects of 4 daily E variables, 12 QTLs, 1 QTLxQTL and 7 QTLxE interactions on the rate (Fig. 1c). The relationship is centered on the mean E values recorded during the MET at the five sites. Dynamic implementation of the model requires daily (*t*) computation of RF_s,g,t_ using the daily values of each of the *i* E factors experienced by genotype *g* at each experimental site (*s*), and NOT the average values over the vegetative phase of each RIL at each experimental site as implemented in the model developed by Bhakta et al. [27].

**Table 1:** Estimated model parameters of differential equation describing the rate of development (RF_sgt_) towards time-to-flowering as a function of QTL genotype operators (TF_g,j_) and environmental factors (f_i,sgt_ = DL, Srad, Tmax and Tmin) at five experimental sites (s)

Variable	Parameter	Estimate	Description
**Center**	μ^c^	0.0235198065831426	Overall Center
**DL**_**sgt**_	α_1_	−0.0015423214758081	Environmental Factors
**Srad**_**sgt**_	α_2_	−0.0000853054955707	
**Tmax**_**sgt**_	α_3_	0.0005966415621148	
**Tmin**_**sgt**_	α_4_	0.0005228804696115	
**TF1**_**g**_	β_1_	0.0009382848209270	QTL Operators
**TF2**_**g**_	β_2_	0.0012733168457595	
**TF3**_**g**_	β_3_	−0.0006810089804720	
**TF4**_**g**_	β_4_	0.0002546737990060	
**TF5**_**g**_	β_5_	−0.0000294375056886	
**TF6**_**g**_	β_6_	0.0005598758808943	
**TF7**_**g**_	β_7_	−0.0004332155371734	
**TF8**_**g**_	β_8_	−0.0002159196193359	
**TF9**_**g**_	β_9_	−0.0004693200551048	
**TF10**_**g**_	β_10_	−0.0002745608987544	
**TF11**_**g**_	β_11_	0.0003463304080702	
**TF12**_**g**_	β_12_	−0.0001723147103969	
**TF1**_**g**_ **× TF2**_**g**_	θ_12_	0.0002794695951983	Interaction: QTL by QTL
**DL**_**sgt**_ **× TF3**_**g**_	γ_13_	−0.0007636661891134	Interaction: Environmental Factor by QTL Operator
**DL**_**sgt**_ **× TF7**_**g**_	γ_17_	−0.0001527383245705	
**DL**_**sgt**_ **× TF12**_**g**_	γ_112_	−0.0001400623472723	
**Tmin**_**sgt**_ **× TF2**_**g**_	γ_42_	−0.0000298150506285	
**Tmin**_**sgt**_ **× TF3**_**g**_	γ_43_	−0.0000818315988969	
**Tmax**_**sgt**_ **× TF5**_**g**_	γ_35_	0.0000980123505664	
**Srad**_**sgt**_ **× TF12**_**g**_	γ_212_	−0.0000274938204046	

Predictor variables are: DL_sgt_ = day length (hours) experienced by genotype *g*^*th*^ on day *t* at site *s;* Srad_sgt_ = solar radiation (Srad, MJ m^−2^ d^−1^) experienced by genotype *g*^*th*^ on day *t* at site *s*; Tmax_sgt_ = maximum temperature (°C) experienced by genotype *g*^*th*^ on day *t* at site *s*; Tmin_sgt_ = minimum temperature (°C) experienced by genotype *g*^*th*^ on day *t* at site *s*; TF1_g_ to TF12_g_ = QTL operators coded as +1 for Calima alleles and − 1 for Jamapa alleles

Thus, day *t* on which genotype *g* first flowers at site *s* is determined by integrating the rate of development function (Fig. 2) over time until it computes a threshold value of 1.0 for *P*_*s,g,t*_ (unitless) according to:

2 $$Ps,g,t={\int}_0^t{RF}_{s,g,t} dt$$

where *P*_*sgt*_ = 0 at *t* = 0 (planting day). The assumption of linearity of E effects on developmental rate is reasonable for environments that do not reach lower or upper limits where the responses are highly nonlinear. This is the main reason for centering the model on the E factors, and also to adequately represent the effect of the multiplicative terms (QTLxQTL and QTLxE) over the range of data in the MET. The dynamic model produced a highly accurate estimation of the TTF phenotype using genetic (QTL) and environmental (E) inputs (Fig. 1c).

### Comparative analysis of model performance

We compared the performances of CROPGRO, the static mixed-effects model developed by Bhakta et al. (2017), and the dynamic model described here for their abilities to simulate the TTF phenotype in the common bean (See Table 2). For this purpose, we identified 123 RILs that had observations at all 5 MET sites. CROPGRO requires the estimation of three model parameters (GSPs) for each RIL to simulate TTF. For this reason, evaluations were carried out independently for each RIL. While crop model parameters had to be estimated for each genotype, mixed-effect model parameters were estimated for the entire population and consequently, data from a total of 815 RIL-site combinations were used for model evaluation.

**Table 2:** Comparative analysis of the performances of Cropgro-Bean, and the Static and Dynamic Mixed-Effects models

Model	Count	MEff.	R^**2**^	R^**2**^_**Adjusted**_	R^**2**^_**Adjusted**_/R^**2**^
Cropgro-Bean^a^	123	0.955	0.961	0.843	0.874
Std	123	0.038	0.032	0.129	0.110
Min	123	0.745	0.845	0.379	0.449
Max	123	0.998	1.000	0.999	0.999
Static M-E	815	0.866	0.867	0.863	0.995
Dynamic M-E	815	0.911	0.912	0.909	0.997

^a^Mean values of performance for Cropgro-Bean; calculations were carried out independently for each RIL that had data at all five MET sites

Model efficiency (Eq. (5)) estimates for CROPGRO averaged 0.955, with a range of 0.745 to 0.998, and a small standard deviation. This suggested that the variation not explained by the model was relatively small compared to the variation in the observed values. Model efficiencies in the static and dynamic models were lower than the CROPGRO average, 0.866 and 0.911, respectively, suggesting that CROPGRO is a better model, on average. However, the poor genetic tractability of CROPGRO parameters reported above, and the wide range of variation in the prediction of TTF by CROPGRO indicated a component of uncertainty and required further assessment.

To conduct a further evaluation of the models we used the R^2^_adjusted_ (Eq. (6)) value because it adjusts for both population size and for the number of model parameters. Accordingly, CROPGRO produced a total of 369 parameter values resulting in 615 simulations. The R^2^_Adjusted_ values of the RILs averaged 0.843 with a range between 0.379 and 0.999. In contrast, the static and dynamic mixed-effects models used only 25 parameter values for the entire populations grown at the five MET sites. Thus, these parameters simulated 817 RIL-site combinations with R^2^_Adjusted_ values of 0.863 for the static and 0.909 for the dynamic models. Factoring in sample size and the number of parameters showed that the average R^2^_Adjusted_ value for CROPGRO simulations represented on average a 0.87 fraction of the R,^2^ with a range of 0.449 to 0.999. In contrast, the R^2^_Adjusted_ values of the static and dynamic mixed-effects models were a 0.99 fraction of their respective R^2^ values indicating that the number of parameters did not artificially increase model performance.

### Validation of the dynamic model

**Fig. 3:**
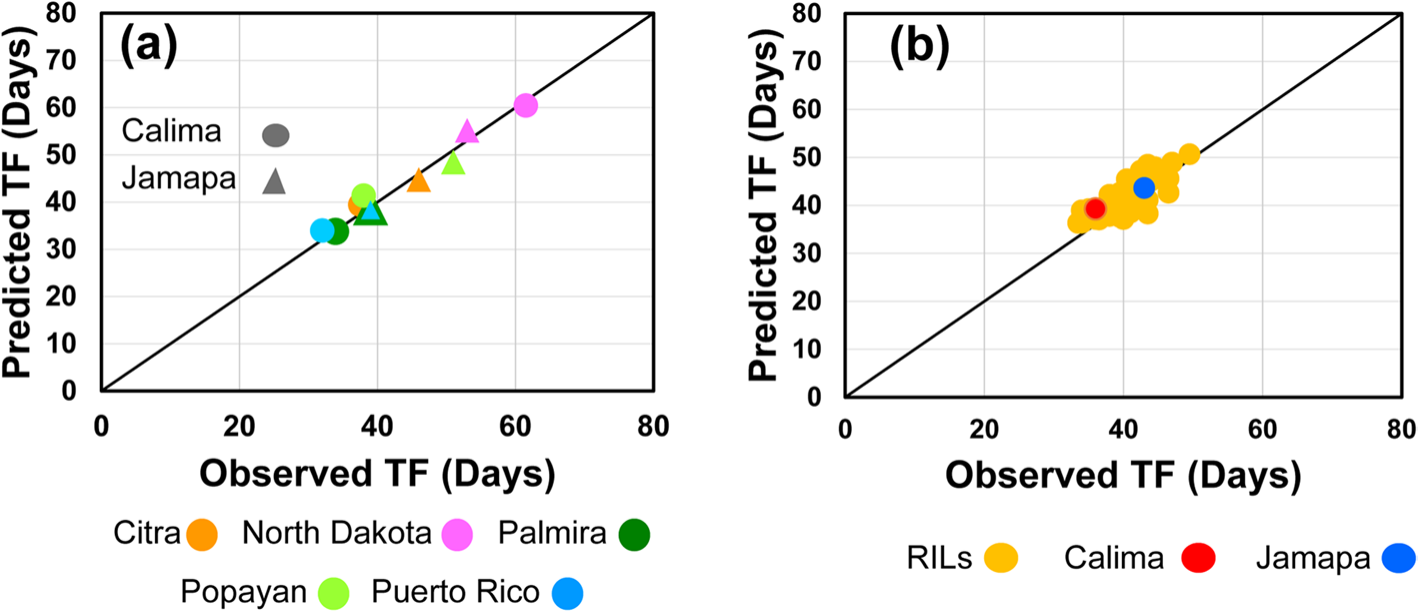
Validation tests of the dynamic simulation model. Observed vs. predicted plots (1:1) of two data sets not involved in generating of the model. **a** Parental genotypes that were grown along the RI family at the five MET sites (Model Efficiency = 0.997; RMSE = 1.54 days; R^2^ = 0.97). **b** A subset of 100 RILs, along with the parents, were grown in Citra, FL in 2016 (Model Efficiency = 0.36; RMSE = 2.44 days; R.^2^ = 0.64)

Validation of the dynamic model was carried out with two independent data sets. The first set comprised the parental genotypes that were grown at the five MET sites but were not included in the construction of the model (Fig. 3a). The high correlation (R^2^ = 0.97) between observed and predicted values indicated that the model performed very well under the environments used for model development but with genotypes (parental) that were not included in its development. The second set included data from a planting of a subset of 100 RILs along with the parents in 2016 in Citra, FL, an environment not used in model development. Model prediction in this trial (Fig. 3b) showed medium performance with an R^2^ value of 0.64. This decrease in model performance is likely due to the slightly higher temperatures than those experienced in the 2012 Citra experiment used for model development. The 2016 Citra results are similar to those from North Dakota suggesting that the model has yet to capture the effect of the interaction between high temperature and long days. However, the root mean squared error for the parental lines in the MET was 1.5 days and 2.4 days that for the 2016 Citra experiment indicating the model has a reliable prediction ability.

### Sensitivity analyses

Sensitivity analyses of the flowering module analyzed the range of combinations of environmental variables (Tmax_*s,t*_, Tmin_*s,t*_, Srad_*s,t*_, and DL_*s,t*_) for different genotypes for each day *t* after planting. Environmental variable ranges were restricted to those observed in the MET, but with small extrapolations in the values to study the overall patterns of effects.

**Fig. 4:**
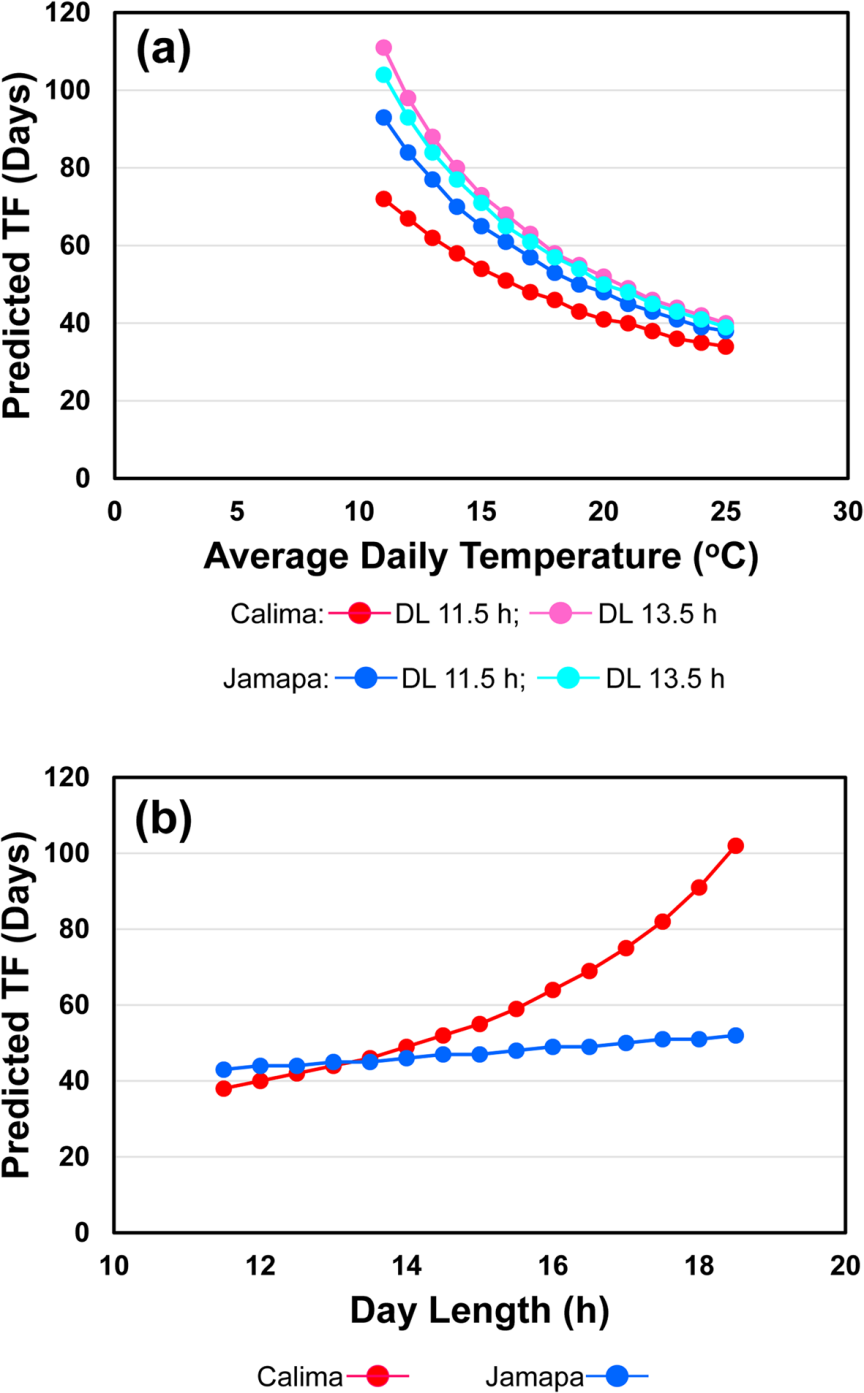
Sensitivity analysis of the Time-to-Flowering module using the parental genotypes: Calima and Jamapa. **a** Simulations of temperature effects on TF according to Eqs. (2) and (3) under two day-lengths. **b** Simulation of day length effects on TF with Tmax and Tmin values of 26 and 18 °C, respectively

Overall, the simulated effect of average daily temperature ((Tmin + Tmax)/2) on TTF was similar for the parental genotypes (Calima and Jamapa), with each displaying the typical curvilinear shape, which is due to the linear effect of temperature on developmental rate (Fig. 4a and b). Under short days (11.5 h), Jamapa’s TTF was longer than Calima’s over the temperature range. Dropping the average daily temperature from 25 °C to 11 °C increased Calima’s TTF from 34 to 72 days (38-day delay), and Jamapa’s TTF from 38 to 93 days (55-day delay). These results indicated that under short days, Jamapa’s TTF is significantly more susceptible to lower temperatures. However, the same temperature drop under a 13.5 h day length increased Calima’s TTF from 40 to 111 days (71-day delay) and Jamapa’s TTF from 39 to 104 days (65-day delay), indicating that under longer photoperiods Calima’s TTF is significantly more susceptible to low temperatures. In fact, no flowering occurred within 200 days at the lowest temperature for days longer than 15 h.

We also conducted day length simulations holding Tmin and Tmax constant at 18 and 26 °C, respectively (Tavg = 22 °C). These simulations showed that increasing day length delays TTF in both parents (Fig. 4b). However, this effect is significantly larger in Calima than in Jamapa. Under an 11.5 h photoperiod, Calima’s TTF was 38 days, only six days earlier than Jamapa’s. Extending the photoperiod to 18.5 h increased Calima’s TTF to 102 days (64-day delay), while Jamapa’s TTF was only delayed by 9 days. These results highlight the photoperiod sensitivity of Calima, which is in agreement with results reported by Bhakta et al. [27] and in this manuscript.

A final set of simulation runs was made to explore how changes in specific QTLs might affect responses to temperature (Fig. S1) and day length (Fig. S2). The response of RIJC366 to temperature was similar to that of Jamapa, but with a TTF about 5 days shorter than Jamapa’s throughout the temperature range. In contrast, RIJC031 was more sensitive to temperature variations than either of the parent genotypes. RIJC031’s TTF increased by 69 days when the average temperature was dropped from 25 to 11 °C, about 50% more than either parent. Transgressive inheritance could be explained by overdominance, but the nature of the progeny excludes this possibility. Epistasis and gene complementarity can also be responsible for transgressive behavior. The analysis identified one epistatic interaction suggesting that most of the behavior can be ascribed to gene complementarity. This is the case in which the two parental genotypes have a subset of QTLs that delay TTF and a subset that shortens it. This is reflected by the sign of the parameter coefficients for each of the 12 QTLs (Table 1 and Fig. 2), some have a positive sign while others have a negative sign (See also the sign of the allele operators in Table S4). RI lines that inherited all the QTL alleles that delay TTF the most are at one extreme of the distribution, and those that inherited QTL alleles that shorten TTF the most are at the opposite extreme. This transgressive behavior was also reported by Bhakta et al. [27].

Day length simulations show the responses of RIJC031 and RIJC366 to be similar to that of Jamapa. However, RIJC366’s TTF was shorter than that of Jamapa throughout the range of day lengths, this transgressive behavior would be similar to the one observed for the temperature response. Simulations were also carried out with synthetic lines modeling the effects of QTLs TF1_g_, TF2_g_, and TF3_g_ on Jamapa (Fig. S3). TF3_g_ has a direct effect on the day length response, whereas TF1_g_ and TF2_g_ have an effect through multiple interactions. The allelic effect at each of these QTL is controlled by the interactions of the allele operator sign (Eq. (3) and the sign of the corresponding coefficient (Fig. 2). The first synthetic genotype, genotype (J-CTF1, 2, 3) was created by replacing in Jamapa its TF1, TF2, and TF3 alleles with the corresponding Calima alleles. Similarly, the second synthetic genotype (J-CTF3) was generated by replacing in Jamapa its TF3 allele with that of Calima. The triple QTL replacement resulted in a modified Jamapa response to photoperiod that was similar to that of Calima. Interestingly, the single replacement with the Calima TF3 allele produced a modified Jamapa response that exceeded that of Calima, a transgressive behavior which could be explained by interactions between the TF3 Calima allele and day length.

## Discussion

Testing the hypothesis that crop model parameters contain genetic information produced mixed results. On the one hand, we detected some QTLs for model parameters controlling the TTF phenotype in CROPGRO, but on the other hand those QTLs did not explain all the genetic variation observed for this trait and did not completely match all the QTLs detected by Bhakta et al. [27]. These results are very similar to those reported by Bogard et al. [16], who detected high predictability (r^2^ = 0.97) of heading time by the wheat-APSIM model, which was reduced significantly (r^2^ = 0.68) when model parameters were turned into linear functions of QTLs. Taken together, these results indicated that not all the information in model parameters is genetically tractable. Crop simulation models were not developed within a genetics framework, but with an exclusive focus on the environmental dependencies of plant processes, while model parameters were devised to represent these dependencies.

The comparative analysis of the models showed that, on average, CROPGRO appears to have a high predictive ability. However, the variability of TTF prediction in the RI family, as shown by the wide range of R^2^_Adjusted_ values (0.379 - 0.999) showed a strong component of unpredictability. This is further underscored by the range in the R^2^_Adjusted_/ R^2^ ratios (0.45 – 0.99). These observations suggest that some model assumptions may not necessarily apply to all genotypes. For instance, most models, including CROPGRO, assume cardinal temperatures are fixed in a species. However, if the range of temperatures for biological activity of individual genotypes vary significantly, then overlooking this type of variation may create challenges for models that do not consider this phenomenon at all.

The results presented in this manuscript clearly indicate that statistical mixed-effects models can be used effectively to predict the phenotype using genotypic (G) and environmental (E) data inputs. Unlike other models, this model was developed without assuming that all genotypes have the same temperature response; furthermore, it included significant and specific GxE interactions. An important difference between the crop model-genetics approach and the mixed-effects approach is as follows. While the crop model approach used derived phenotypes (estimated model parameters) to obtain QTL information, the mixed-effects model used QTL information obtained from genetic analysis of observed phenotypes. These results also demonstrated that the overall approach used in many crop models for computing rate of progress toward first flowering can be used in a statistical approach in which G, E, and GxE effects are considered. The increase in model accuracy of the dynamic model (R^2^_Adjusted_ = 0.909) over the static model of Bhakta et al. [27] (R^2^_Adjusted_ = 0.863) can be ascribed to the use of daily E input values instead of average E factor values over a period of time.

Phenology modules play a key role in crop models because they set the rate of development for the crop, which can significantly affect productivity by altering the dynamics of assimilate partitioning throughout the crop’s life cycle. However, phenology modules are typically difficult to parameterize because they are commonly affected by GxE interactions. Thus, the inclusion of specific GxE interactions in the dynamic mixed-effect TTF model makes it a potential key module component of a comprehensive crop model. Results of the sensitivity analyses with the parental genotypes and few selected RI lines along with the validation exercises at the five experimental MET sites provide strong support for the TTF dynamic model, but the validation exercise obtained with the 2016 Citra data (Fig. 3b) indicated that the effect of low temperatures in combination with long days still need to be fully captured by the model.

Genomic selection [30] has been adopted by plant breeding programs world-wide because it is an affective methodology to predict the phenotype, including time-to-flowering [40, 41] and in combination with artificial neural networks [42]. However, there are fundamental differences between genomic selection and QTL analysis. The main objective of genomic selection is genetic gain, which is attained by fitting all polymorphic markers across the entire genome in a linear model that explains the phenotype. In other words, the phenotype of an individual is predicted as the sum of the breeding values of all segregating markers. This is done without significance testing for any specific marker, or by targeting any specific genes. Genomic selection has proven more effective than marker assisted selection particularly when dealing with quantitative traits controlled by large numbers of genes, each with a small contribution to the trait [43] and has also been integrated with crop modeling to predict GxE interactions [44]. In contrast, QTL analysis aims to identify genes that affect the phenotype. This approach is effective in the analysis of quantitative traits controlled by relatively few genes with major contributions to the phenotypes as is the case of developmental transitions such as time-to-flowering. Typically, QTLs are tracked through linked markers before their identity is clearly determined. Thus, incorporating QTL information into a dynamic model creates to possibility of eventually constructing a mechanistic gene-based model for different plant processes.

The construction of a dynamic mixed-effect model that uses G and E data as sole inputs, as described above, could be applied to other plant processes in crop species in general. This raises the possibility of developing dynamic gene-based crop simulation models (DGCSM) capable of integrating information from interconnected phenology and growth process-oriented modules. Growth process modules trace resource acquisition of different organs. For example, a leaf area expansion module could integrate input from sub-modules for rate of leaf appearance, individual leaf area expansion, branch appearance, and similar branch sub-modules. This approach accentuates the increased granularity required by DGCSM, a characteristic that makes the phenotype more genetically tractable and reduces equifinality problems in phenotype prediction; for instance, two genotypes with the same leaf area – one with few large leaves and the other with many small leaves. Furthermore, determining the identities of QTLs can create a powerful connection between DGCSM and dynamic gene regulatory networks, and therefore establish a G2P bridge across scales of time and space.

Current crop models can be gradually converted into DGCSM by replacing existing process model components with modules that incorporate G, E, and GxE relationships. It represents an innovative approach that combines genetics and modeling to increase both prediction effectiveness and understanding of genetic effects on biological processes. We have integrated a stand-alone two-module dynamic simulation program into the DSSAT CROPGRO model [45]; see also Supplementary File). The first module computes the daily rate of development according to the fitted function (Table 1; Fig. 2) and requires the QTL allele operators of individual genotypes and daily environmental values (Tmax_*i*_, Tmin_*i*_, Srad_*i*_, and DL_*i*_). The second module integrates the daily rates over time according to Eq. (2) to predict time to first flower. The code can be extended to a wider genotypic base and environmental range after proper estimation procedures.

**Fig. 5:**
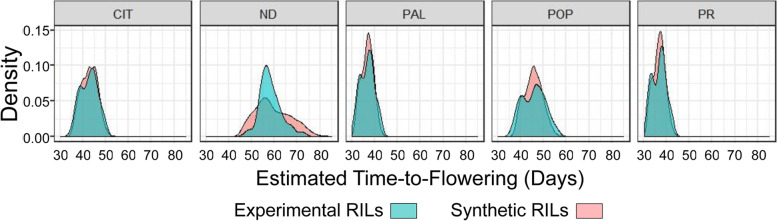
Density plots of Time-to-Flowering estimate of the RI family at the five experimental sites. Frequency distribution of the experimental RI family (Green; *n* = 188) and the synthetic RI family comprising all 4096 possible allelic combinations of the 12 QTLs as simulated by the dynamic module. These plots indicate that multiple allelic combinations can attain the same phenotype providing a choice of convenience to plant breeding programs

To illustrate the usefulness of DGCSM in plant-breeding, we simulated the phenotypes of multiple QTL allelic combinations grown at each one of the five experimental sites. These are all possible allelic combinations (2^12^ = 4096) of the 12 QTL segregating in our experimental RI family (Fig. 5). Such simulation enables evaluation and selection of suitable allelic combinations that produce a desirable TTF phenotype in specific environments, which may include those projected for climate change. DGCSM can expedite genetic progress by reducing the need for expensive MET. Furthermore, as an aid in precision breeding, DGCSM can be turned into expert systems to search the gene space (QTL database) to create suitable ideotypes [46, 47]. Finally, projected increases in human population and climate change highlight the urgency with which worldwide food security needs to be addressed [48]. DGSCM in conjunction with climate models could assist policy decision makers in developing information about food supply futures to improve worldwide stability.

## Conclusions

We have shown that although estimated parameter values of traditional crop simulation models can efficiently simulate the phenotype, extraction of genetic information from those parameters remains a difficult challenge making it difficult to convert those parameters into functions of the genotype. More challenging yet is the extraction of the GxE interactions effects these parameters may have. Our comparative analysis has shown that the dynamic mixed-effects model can more effectively capture the G and GxE interaction components of variation to predict the phenotype. Also, unlike the previously developed static model, the dynamic model can show progress towards the timing of a developmental transition (TTF in this case) in real time because it is responsive to the daily environmental fluctuations.

The dynamic mixed-effects approach can be used to model other plant processes, not only in beans, but in other species as well. A TTF module has already been incorporated into BeanGro in the DSSAT system [45], which underscores the possibility of converting traditional crop simulation models into DGCSMs. The simulation of all possible QTL combinations indicates that dynamic mixed effects models can be used to design ideotypes adapted to specific environments, including those predicted by climate change. Furthermore, determining the identity of QTLs will facilitate connecting the genes that mold the phenotype with gene regulatory networks, which may lead to more rational and effective genetic manipulation of crop species.

## Methods

### Plant material

The TTF phenotypes were collected from a RI family (n = 188, F_11:14_) that was obtained from a bi-parental cross between a Mesoamerican and an Andean bean cultivar [27]. Jamapa is a small black seeded bean cultivar from Mexico with an indeterminate growth habit and insensitive to photoperiod, whereas Calima is a large seeded and mottled Colombian bean cultivar with a determinate growth habit and sensitivity to photoperiod. The linkage map derived from this population was described previously [32] and the genotype data for the population can be found online at https://figshare.com/s/50d1ddcaf8c04026dd4c.

### Experimental sites

Five geographical locations were selected to provide contrasting temperature and photoperiod conditions (Table S1). These included Citra, Florida (CIT); Prosper, North Dakota (ND); and Isabela, Puerto Rico (PR) in the United States; the Colombian sites Palmira, (PAL) and Popayan (POP) near the equator provided short days, and their altitudinal difference (800 m) a temperature differential. Daily weather data from these sites are available in Additional File 1. The RI family was planted using a row-column experimental design model, which was used for spatial correction as needed and for an ANOVA that was used to calculate heritabilities at each site and across sites as described by Bhakta et al. [27].

### Parameter estimation of genotype specific parameters (GSPs) for predicting anthesis day after planting (ADAP)

GSPs were estimated to predict ADAPs in the DSSAT CROPGRO-Dry Bean model (v. 4.5). These were planting-to-emergence (PLEM, thermal days), emergence-to-flowering (EMFL, photothermal days), and the slope of the relative response of development to photoperiod with time (photoperiod sensitivity, PPSEN, h^−1^). These GSPs were estimated for each genotype (parental and RILs) using a stepwise Markov chain Monte Carlo estimation approach as described previously [19], but with minor changes. Briefly, PLEM was estimated first for each genotype across all five sites using emergence day after planting (EDAP) as the target output. For PLEM, the minimum and maximum values of the normal distributions for generating the proposal distributions were set to 2 and 20 thermal days, respectively. Next, EMFL was estimated using ADAP as the target output for each genotype across four of the five sites (i.e., all sites excluding ND) and the minimum and maximum distribution values were set to 20 and 40 photothermal days, respectively. Finally, PPSEN was estimated for each genotype with ADAP in ND as the target output (long-day site) with the minimum and maximum distribution values set to 0.001 and 0.500 h^−1^, respectively. Based on trace plots observed for all parameters, 3000 iterations of GSP sampling (burn-in length set at 1000 iterations) were sufficient for convergence. The critical short-day length (CSDL) below which reproductive development progresses with no day length effect (for short day plants), was set to the default value of 12.17 h.

### QTL mapping

QTLs that control GSPs associated with the TTF phenotype were mapped using composite [49] and multiple [50] interval mapping as described elsewhere [27]. The search for QTLs was performed with a window size of 5 cM, while a 50 cM distance was used as the minimum cofactor distance in the composite interval mapping scan. Threshold LOD values for QTL detection were determined at significance level of 0.05 using results obtained after 1000 random permutations.

### Development of the mixed-effects and dynamic models

This model was derived from the one developed by Bhakta et al. [27]. However, instead of using TTF (days) as the observed response, we modeled the rate of development towards flowering (1/TTF (days)). The generic structure of the model based in *i* environmental variables and *j* QTL operators is:

4 $${RF}_{sgt}={\mu}^c+\sum_i{\alpha}_i\times {f}_{i, sgt}^c+\sum_j{\beta}_j\times {TF}_{j,g}+\sum_j\sum_{j\ast >j}{\varTheta}_{jj\ast}\times {TF}_{j,g}\times {TF}_{j\ast, g}+\sum_i\sum_j{\gamma}_{ij}\times {f}_{i, sgt}^c\times {TF}_{j,g}+{e}_{sgt}$$

where *RF*_*sgt*_ (= *dP/dt*) is the rate of development for the genotype *g* on day *t* at site *s; μ*^*c*^ is the center, and *α*_*i*_, *β*_*j*_, *ϴ*_*jj*∗_, and *γ*_*ij*_ are the model parameters to fit. In addition, the predictor variables are: ${f}_{i, sgt}^c$ for the centered environmental factors, *TF*_*j,g*_ for the QTL operators, and their two-way interactions *TF*_*j,g*_ *× TF*_j*,g_ and *f*_*i,sgt*_×*TF*_*j,g*_. Finally, *e*_sgt_ is the random residuals assumed to be a multivariate Normal distribution of the form ***e*** ~ MVN(**0**, **R**), where **R** is a matrix of variance-covariance of unstructured form included to model the correlated nature of the observations belonging to the same genotype across sites. Note that the QTL allele operators for each genotype are coded as +1 for Calima alleles and − 1 for Jamapa alleles. The above model was fitted using the strategy proposed by Malosetti et al. [26], and further details were presented in Bhakta et al. [27]. The final model estimated the parameters for *i* = 4 environmental variables, *j* = 12 TF QTLs, a single G-by-G interaction and seven G-by-E interactions; the environmental variables represent the dynamic conditions under which the model operates. In a second step, the TTF of each RIL at each of the five experimental sites was predicted by first estimating the daily rates of development towards flowering (1/TTF (days)) by using as inputs the allelic makeup of the RIL at each of the 12 QTLs and the daily values of the four environmental variables (day length, solar radiation, and daily maximum and minimum temperatures). The TTF was identified when the daily integration of the daily rates reached the value of 1.0.

A few general assumptions have been made for the dynamic mixed-effects model. The first is that responses to environmental effects are linear for any RIL within the range recorded during the MET for those variables (Tmax_sgt_, Tmin_sgt_, SRad_sgt_ and DL_sgt_). The model does not account for known nonlinearities in responses to these environmental variables that may occur in other environments. Likewise, it is assumed that there is a linear response to the genetic factors (12 QTLs) segregating in the bi-parental progeny. The dynamic model also assumes that the daily developmental rate is controlled by the allelic makeup at the 12 QTLs of each genotype, and the daily weather conditions to which each genotype is exposed. This dynamic model fully applies to the bi-parental population for which it was constructed. It is also understood that the operational environmental domain of the model could be extended through the adoption of non-linear mixed-effect models, and that the versatility of such model within the crop species could be increased through the discovery of additional loci and alleles.

### Implementation of the dynamic mixed-effects model

A two-part code for the statistical package R was written (Supplementary File) to carry out the procedures described above using the information contained in two additional files. Additional file 1 contains ten fields with the following headings: **SrNO**: Serial Number; **Site**: CIT = Citra, FL; ND=North Dakota, PAL = Palmira, Colombia; POP=Popayan, Colombia; PR = Puerto Rico; **Year:** The year in which the experiment was carried out – 2011 or 2012; **DOY**: Sequential day number starting with day 1 on January 01, which is also known as the Julian date; **DAP:** Number of calendar days after planting; **Srad:** Solar radiation in MJ m^−2^ d^−1^; **DayLhr:** Day length in hours; **Tmax:** Daily maximum temperature in degrees Celsius, ^o^C; **Tavg:** Daily average temperature in degrees Celsius, ^o^C; **Tmin:** Daily minimum temperature in degrees Celsius, ^o^C.

The *‘R1data_weatherDAFtoFF.txt*’ file contains 19 fields with the following headings: **RIL:** The identifier of each RIL from the [Jamapa X Calima] cross; **Site:** Experimental site – CIT = Citra-FL; ND = North Dakota, PAL = Palmira-Colombia, POP = Popayan-Colombia; PR = Puerto Rico; **R1:** Number of calendar days when first anthesis was detected; **TF1 to TF12** (TTF QTLs in the RIL population); **Srad**_**m**_: Average solar radiation between planting and day of first anthesis for each RIL in MJ m^−2^ d^−1^; **DL**_**m**_: Average day length (h) between planting and day of first anthesis for each RIL; **Tmin**_**m**_: Average minimum temperature between planting and day of first anthesis for each RIL in degrees Celsius, ^o^C; **Tmax**_**m**_: Average maximum temperature between planting and day of first anthesis for each RIL in degrees Celsius, ^o^C. The TF QTLs and their chromosome and map positions (cM) according to the linkage map we constructed previously [32] are as follows: TF1, TF2, TF3 and TF4 (Chrom1, 22.1, 42.1, 58.8 and 70.0), TF5 and TF6 (Chrom3, 38.2 and 49.2), TF7 (Chrom4, 42.2), TF8 (Chrom6, 31.3), TF9 and TF10 (Chrom7, 11.7 and 98.7), TF11 and TF12 (Chrom11, 2.1 and 9.3). The numbers in the fields for TF1 to TF12 represent the operators for the Calima (+1) and Jamapa (−1) alleles.

In Part 1, the code estimates parameters for the mixed-effects model. Parts 2a and 2b constitute the dynamic components of the model. The Part 2a section estimates the daily rates of development towards flowering and an integrator adds up the daily progress until it reaches a value of greater than 1.02 for any given RIL at any given site. Part 2b calculates the day of flowering by interpolation using the integrated values flanking the value of 1.0. In addition to predicting TF for the experimental population, the code was included to do the same with a synthetic RIF that included all possible allelic combinations of the 12 TF QTLs. To work with the synthetic family, the first line of code for r1 must be turned into a comment and the following line must be activated by removing the comment symbol. After this change, the program will read the data in Additional File 3 instead of the data in Additional File 2.

### Model evaluation

Model efficiency (ME) is a measure of the predictive skill of the model. It expresses the model error based on a predictor. At one extreme, if the prediction is a perfect one, then the efficiency is 100%. However, if the average observation is used as the predictor and there is no difference between the average and the predicted value, then the efficiency is zero.

5 $$ME=1-\left[\frac{\sum_{i=1}^n{\left({y}_i-{\hat{y}}_i\right)}^2}{\sum_{i=1}^n{\left({y}_i-\overline{y}\right)}^2}\right]$$

where *y*_*i*_ is the _*i*_^th^ observation, ${\hat{y}}_i$ is the _*i*_^th^ predicted value, and $\overline{y}$ is the mean of the observed values.

The adjusted R^2^ is a modified value of the coefficient of determination that is adjusted for the number of predictors in the model and the size of the population. The R^2^adjusted decreases when the addition of parameters does not improve the fit of the model.

6 $${R}_{Adjusted}^2=1-\left[\frac{\left(1-{R}^2\right)\left(n-1\right)}{\left(n-k-1\right)}\right]$$

where R^2^ is the coefficient of determination, *n* is the number of samples and *k* is the number of parameters in the model.

### Development of gene-based time-to-flowering plug-in module

A computer program in FORTRAN (Supplementary File) was developed to produce a module for the dynamic simulation of the TTF, and to demonstrate the sensitivity of its prediction ability to both genetic and environmental variations. This dynamic module was designed to easily integrate into an existing ecophysiology model for beans, which simulates seasonal biomass growth, the timing of phenological events (including flower appearance), and final total and grain biomass [13, 51].

This module has a structure that is similar to the dynamic module described above and integrates two sub-modules. The *Rate of Flowering Sub-Module* contains the daily rate of development Equation (Fig. 2, Eq. (3)), which computes both environmental and genetic input information. The *Driver Main Flowering Sub-Module* specifies the daily environmental variables and the genetic variables (QTL allele operators) for any particular genotype and passes them as inputs to the *Rate of Flowering Sub-Module* where the dynamic model is programmed. In turn, the daily rate and cumulative development, represented by *SumRF*_*i,j*_, is passed back to the driver main program. The module was designed this way so that it can be inserted into some other program, including a more comprehensive ecophysiology model like the CROPGRO-Bean model [13]. The integrated operation of the sub-modules allows the simulation of TTF for different combinations of daily environmental conditions to create a type of sensitivity analysis to demonstrate some of the important variations that occur across environments for selected genotypes. For instance, the second sub-module could be made to read actual daily weather data and genetic variables for a simulation of one situation. Instead, this module specifies constant daily weather values, which are preselected to study how variations in any one variable (such as day length or temperature) influence rate of progress and time to first flower, a simple sensitivity analysis. This module also writes result files. The computer code and a brief summary of its modules (Rate of Flowering Sub-Module and Driver Main Flowering Sub-Module) can be found in the Supplementary File.

### Simulation study procedures

A simulation analysis was performed to explore variations among the lines as affected by the two most important environmental conditions in which the plants are grown. The gene-based time to flower model (RF Module and Driver Module) simulated the behavior of two RILs and the two parents of the RIJC population. Neither of the two parents were included in the bean MET dataset used to fit the model. The first RIL (RIJC031) was selected at random from the RILs in the population and the second RIL (RIJC366) was selected to have half of its QTL alleles different from the first one. These were arbitrarily selected to compare with the simulated responses of the two parents. Table S4 lists the QTL allele operators for these genotypes that were used to explore model responses to temperature and day length.

These four genotypes (Calima, Jamapa, RIJC031, and RIJC366) were simulated over a range of temperatures that represented the range that occurred across the five sites. For each simulation, day length and solar radiation were held constant so that only daily maximum and minimum temperatures were changed for each run but held constant for the duration of the simulation. The difference between daily maximum and minimum temperature was arbitrarily held constant at 8 °C. This resulted in a range of average daily temperatures that varied from 11 to 25 °C. For these simulations of temperature responses, day length was held constant at its mean value of 12.8631 h and solar radiation was held constant at its mean value of 18.2719 MJ m^−2^ d^−1^.

The four genotypes were then simulated 15 different times, varying day length for each one. Day length in each run was held constant whereas day length was varied among runs (between 11.5 and 18.5 h) (see the Driver Module). For the results presented for variations in day length, daily maximum and minimum temperatures were held constant at 26 °C and 18 °C, respectively; daily solar radiation was also held constant for all of these runs at its mean value from the multiple environment trials (18.2719 MJ m^−2^ d^−1^). Results presented below were all based on simulations of the model. However, since the model described 91% of the variability in the observed data, they represent responses based on data, not on prior assumptions about response functions.

## Supplementary Information

**Additional file 1: Table S1**. Geographical and environmental characteristics of the five experimental sites. **Table S2**. Genotype-specific parameter (GSP) values. **Table S3**. Summary of QTL mapping results.**Table S4**. Allele operators of the TF QTLs. **Figure S1**. Temperature simulations generated with the dynamic gene-based time-to-first-flower bean model – implementation of Eqs. (2) and (3). **Figure S2**. Photoperiod simulations generated with the dynamic gene-based time-to-first-flower bean model – implementation of Eqs. (2) and (3). **Figure S3**. Photoperiod simulations of real and synthetic genotypes created through substitutions of QTL alleles. **Supplementary Text**. Flowering Rate Model- R code. Rate of Flowering Sub-Module – Rate of Progress toward First Flower. Driver Sub-Module. Weather_daily.csv. RIL_R1sata_weatherDAPtoFF.csv. AllQTLcombo.csv.

## Data Availability

Computer codes are available in the Supplementary Materials file, and observational and synthetic data in Additional file 1, which have been uploaded to the figshare database repository (10.6084/m9.figshare.19692628).

## Abbreviations

ADAP: Anthesis Days After Planting
CIT: Citra, Florida
CSD: Critical Short Day
DGCSM: Dynamic Gene-based Crop Simulation Models
DL: Day Length
E: Environment
EDAP: Emergence Days After Planting
EM-FL: Emergence-to-Flowering
G: Genotype
G2P: Genotype-to Phenotype
GSP: Genotype-Specific Parameter
GWAS: Genome-Wide Association Study
LOD: Log of the Odds
ME: Model Efficiency
MET: Multiple Environment Trial
MVN: Multi Variate Normal distribution
ND: Prosper, North Dakota
PAL: Palmira, Colombia
PL-EM: Planting-to-Emergence
POP: Popayan, Colombia
PPSEN: Photoperiod Sensitivity
PR: Isabela, Puerto Rico
QTL: Quantitative Trait Locus
RF: Rate of Development Towards Flowering
RI: Recombinant Inbred
SRAD: Daily Solar Radiation
Tmax: Maximum Daily Temperature
Tmin: Minimum Daily temperatures
TTF: Time-to-Flowering
