# Supplementary material for: Dynamic QTL-based ecophysiological models to predict phenotype from genotype and environment data
Source: BMC Plant Biol. 2022 Jun 6;22:275. doi: 10.1186/s12870-022-03624-7 (PMC9169398; doi:10.1186/s12870-022-03624-7)
Supplement: Supplementary file 1 — Additional file 1: Table S1. Geographical and environmental characteristics of the five experimental sites. Table S2. Genotype-specific parameter (GSP) values. Table S3. Summary of QTL mapping results.Table S4. Allele operators of the TF QTLs. Figure S1. Temperature simulations generated with the dynamic gene-based time-to-first-flower bean model – implementation of Eqs. (2) and (3). Figure S2. Photoperiod simulations generated with the dynamic gene-based time-to-first-flower bean model – implementation of Eqs. (2) and (3). Figure S3. Photoperiod simulations of real and synthetic genotypes created through substitutions of QTL alleles. Supplementary Text. Flowering Rate Model- R code. Rate of Flowering Sub-Module – Rate of Progress toward First Flower. Driver Sub-Module. Weather_daily.csv. RIL_R1sata_weatherDAPtoFF.csv. AllQTLcombo.csv. [file 12870_2022_3624_MOESM1_ESM.docx]

**SUPPLEMENTARY INFORMATION**

**Title: Dynamic Gene-Based Ecophysiological Models to Predict Phenotype from Genotype and Environment Data**

***THEORETICAL AND APPLIED GENETICS***

**Authors: C. Eduardo Vallejos^1,2*^, James W. Jones^3^, Mehul S. Bhakta^1,5^, Salvador A. Gezan^4^, Melanie J. Correll^3^**

**Affiliations:**

^1^Horticultural Sciences Department, University of Florida, Gainesville, FL 32611, USA

^2^Plant Molecular and Cellular Biology Graduate Program, University of Florida, Gainesville FL 32611, USA

^3^Department of Agricultural and Biological Engineering, University of Florida, Gainesville FL 32611, USA

^4^School of Forest Resources and Conservation, University of Florida, Gainesville, FL 32611, USA

^5^Current address: Bayer Crop Science, 700 Chesterfield Parkway, West Chesterfield, MO 63017, USA

^6^Current address: VSN International, Hemel Hempstead, United Kingdom

^*^Corresponding author. Email: [vallejos@ufl.edu](mailto:vallejos@ufl.edu)

**Supplementary Figures**

- **Fig. S1 Temperature simulations generated with the dynamic gene-based time-to-first-flower bean model – implementation of Eqs. [2] and [3].**
- **Fig. S2 Photoperiod simulations generated with the dynamic gene-based time-to-first-flower bean model – implementation of Eqs. [2] and [3].**
- **Fig. S3 Photoperiod simulations of real and synthetic genotypes created through substitutions of QTL alleles.**

**Supplementary Tables**

- **Table S1 Geographical and environmental characteristics of the five experimental sites.**
- **Table S2 Genotype-specific parameter (GSP) values.**
- **Table S3 Summary of QTL mapping results.**
- **Table S4 Allele operators of the TF QTLs.**

**Supplementary Text**

- **Flowering Rate Model- R code**
- **Rate of Flowering Sub-Module – Rate of Progress toward First Flower.**
- **Driver Sub-Module.**

**Supplementary Data Files**

- **Weather_daily.csv**
- **RIL_R1sata_weatherDAPtoFF.csv**
- **AllQTLcombo.csv**


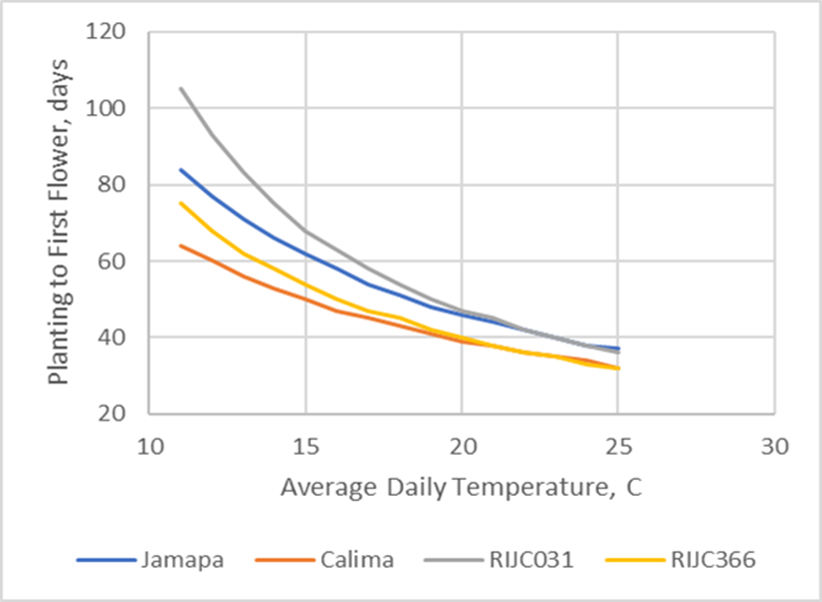


**Fig. S1. Temperature simulations generated with the dynamic gene-based time-to-first-flower bean model – implementation of Eqs. [2] and [3].** The two parental genotypes Calima and Jamapa and two selected RILs shows different temperature dependencies. Notice the simulated transgressive behavior of RIJC031, which is in accordance with the observed data.


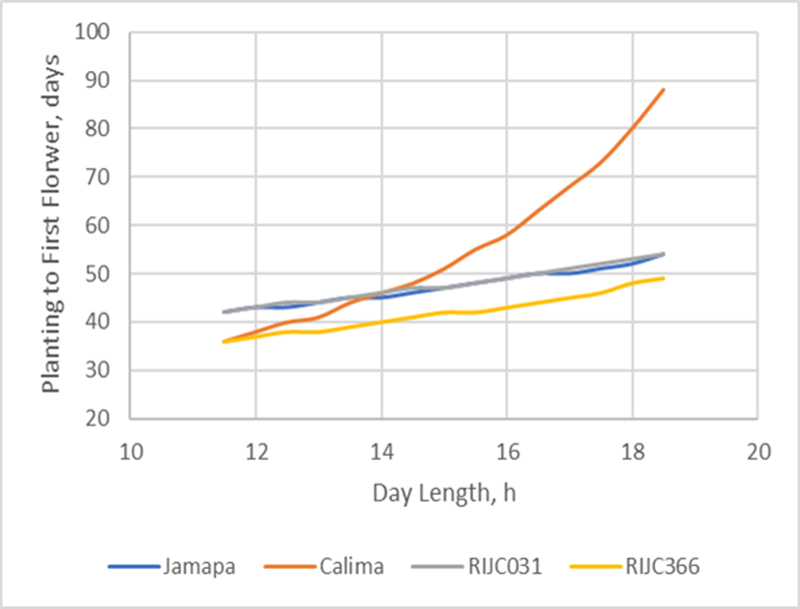


**Fig. S2. Photoperiod simulations generated with the dynamic gene-based time-to-first-flower bean model – implementation of Eqs. [2] and [3.** The two parental genotypes Calima and Jamapa and two selected RILs display different photoperiod sensitivities Notice the simulated transgressive behavior of RIJC366, which parallels the observed data.

**
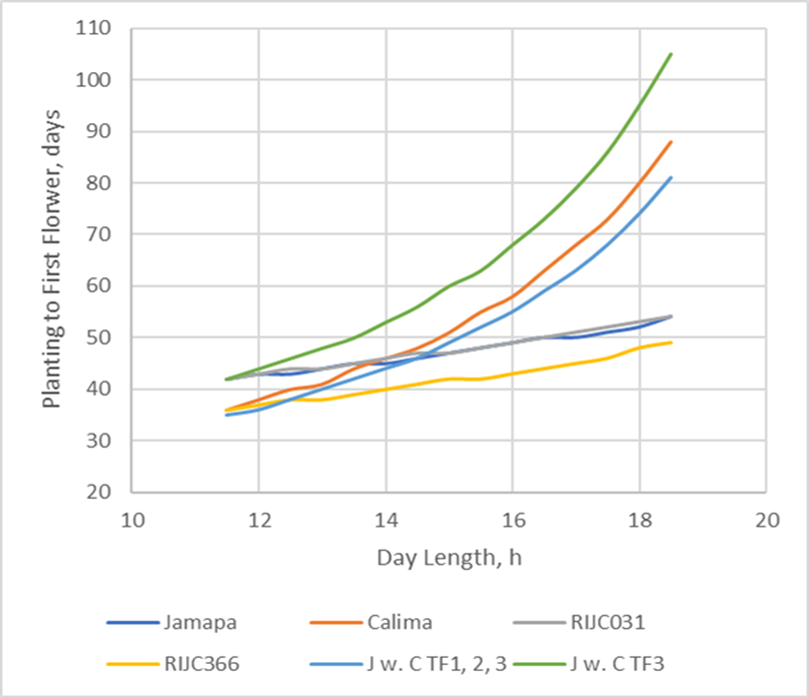
**

**Fig. S3. Photoperiod simulations of real and synthetic genotypes created through substitutions of QTL alleles.** Results for the two parental genotypes Calima and Jamapa, two selected RILs and two synthetic lines. The response labeled “J w. CTF3” corresponds to a synthetic Jamapa genotype by substituting its TF3 allele with the Calima allele, which confers high sensitivity to long days. This single substitution conferred photoperiod sensitivity to Jamapa. Similar allelic replacement at two additional QTLs (TF1 and TF2), as shown by the response labeled “J w. C TF1,2,3”, increased the photoperiod sensitivity directly through TF3 and indirectly through interaction terms with the other QTLs.

| **Table S1.** **Geographical and environmental characteristics of the five experimental sites**. The recombinant inbred family was phenotyped for time-to-flowering at these sites. | | | | | | | | |
| --- | --- | --- | --- | --- | --- | --- | --- | --- |
| **Experimental Site** | **Site ID** | **MASL^a^** | **Latitude** | **Longitude** | **Day-Length**  **range (h)^b^** | **Solar Rad.^c^**  **MJ m^-2^ d^-1^** | **T_max_ (^o^C)^c^** | **T_min_ (°C)^c^** |
| Prosper, ND, USA | ND | 280 | 46° 53’ N | 97° 01’ W | 15:20 - 15:53 | 20.92 | 26.9 | 13.2 |
| Citra, FL, USA | CIT | 31 | 29° 24’ N | 82° 06’ W | 12:30 - 13:30 | 20.30 | 29.4 | 14.3 |
| Citra, FL, 2016^d^ |  |  |  |  | 12:04 - 13:40 | 20.97 | 28.8 | 16.9 |
| Isabela, PR, USA | PR | 128 | 18° 28’ N | 61° 01’ W | 11:30 - 12:35 | 20.82 | 28.1 | 19.7 |
| Palmira, Colombia | PAL | 1000 | 03° 32’ N | 76° 18’ W | 11:56 - 11:58 | 14.17 | 28.2 | 19.6 |
| Popayan, Colombia | POP | 1800 | 02° 26’ N | 76° 36’ W | 12:08 - 12:11 | 15.14 | 24.6 | 14.0 |
| ^a^Meters Above Sea Level. ^b^During growing period. ^c^Average during the growing period. ^d^Ctira 2016 data was used for validation purposes. | | | | | | | | |

| **Table S2 Genotype-specific parameter (GSP) values.** PL-EM (Planting to emergence), EM-FL (Emergence to flowering) and PPSEN (Photoperiod sensitivity) are GSPs associated with time-to-flowering in the DSSAT Bean-CROPGRO model. GSPs for the parents and the RIF were estimated via inverse modeling. | | | | | | | |
| --- | --- | --- | --- | --- | --- | --- | --- |
| **Genotypes** | **PL-EM** | **EM-FL** | **PPSEN** | **Genotypes** | **PL-EM** | **EM-FL** | **PPSEN** |
| CALIMA | 5.75 | 21.7 | 0.129 | RIJC0081 | 3.91 | 27.9 | 0.045 |
| JAMAPA | 4.57 | 30.3 | 0.027 | RIJC0082 | 4.79 | 27.3 | 0.039 |
| RIJC0003 | 4.79 | 21.1 | 0.131 | RIJC0129 | 4.79 | 27.7 | 0.036 |
| RIJC0004 | 5.76 | 28.6 | 0.053 | RIJC0130 | 4.79 | 27.2 | 0.019 |
| RIJC0005 | 4.73 | 23.7 | 0.135 | RIJC0131 | 3.92 | 23.9 | 0.080 |
| RIJC0006 | 3.97 | 21.6 | 0.111 | RIJC0135 | 4.81 | 31.4 | 0.025 |
| RIJC0007 | 6.86 | 27.6 | 0.080 | RIJC0136 | 3.90 | 28.8 | 0.043 |
| RIJC0008 | 4.79 | 22.1 | 0.162 | RIJC0137 | 3.90 | 23.2 | 0.125 |
| RIJC0009 | 3.97 | 29.6 | 0.043 | RIJC0138 | 3.90 | 28.4 | 0.041 |
| RIJC0011 | 6.76 | 32.4 | 0.071 | RIJC0140 | 4.88 | 23.0 | 0.147 |
| RIJC0012 | 4.73 | 22.8 | 0.122 | RIJC0141 | 5.73 | 28.2 | 0.050 |
| RIJC0013 | 4.74 | 22.9 | 0.098 | RIJC0142 | 3.94 | 33.7 | 0.058 |
| RIJC0014 | 5.78 | 22.6 | 0.142 | RIJC0144 | 5.88 | 29.6 | 0.068 |
| RIJC0015 | 3.90 | 22.9 | 0.100 | RIJC0145 | 4.79 | 29.5 | 0.041 |
| RIJC0016 | 4.60 | 31.5 | 0.042 | RIJC0146 | 4.73 | 27.7 | 0.050 |
| RIJC0017 | 4.79 | 28.8 | 0.112 | RIJC0147 | 5.74 | 31.0 | 0.068 |
| RIJC0019 | 5.79 | 25.7 | 0.088 | RIJC0148 | 4.79 | 25.9 | 0.124 |
| RIJC0020 | 4.79 | 23.6 | 0.060 | RIJC0149 | 5.77 | 28.2 | 0.098 |
| RIJC0021 | 4.60 | 23.9 | 0.051 | RIJC0150 | 5.82 | 23.9 | 0.081 |
| RIJC0022 | 5.75 | 31.0 | 0.087 | RIJC0151 | 5.86 | 27.8 | 0.060 |
| RIJC0024 | 6.10 | 28.7 | 0.161 | RIJC0201 | 4.69 | 24.0 | 0.056 |
| RIJC0025 | 5.79 | 26.9 | 0.103 | RIJC0202 | 5.91 | 31.3 | 0.117 |
| RIJC0027 | 4.60 | 27.0 | 0.104 | RIJC0203 | 4.79 | 27.6 | 0.057 |
| RIJC0029 | 4.79 | 22.0 | 0.095 | RIJC0204 | 5.83 | 28.1 | 0.051 |
| RIJC0032 | 6.26 | 27.9 | 0.075 | RIJC0205 | 4.79 | 26.1 | 0.051 |
| RIJC0045 | 5.74 | 24.8 | 0.137 | RIJC0206 | 3.90 | 27.8 | 0.059 |
| RIJC0046 | 4.99 | 26.9 | 0.057 | RIJC0207 | 3.90 | 27.9 | 0.075 |
| RIJC0047 | 4.81 | 22.2 | 0.141 | RIJC0208 | 3.90 | 33.3 | 0.071 |
| RIJC0048 | 4.79 | 29.1 | 0.057 | RIJC0209 | 5.82 | 29.0 | 0.061 |
| RIJC0059 | 4.79 | 28.1 | 0.104 | RIJC0210 | 4.79 | 28.1 | 0.086 |
| RIJC0061 | 4.79 | 30.1 | 0.082 | RIJC0212 | 5.43 | 25.6 | 0.115 |
| RIJC0065 | 4.79 | 26.9 | 0.064 | RIJC0213 | 5.43 | 22.1 | 0.153 |
| RIJC0066 | 5.75 | 23.5 | 0.155 | RIJC0214 | 5.73 | 24.0 | 0.120 |
| RIJC0067 | 4.79 | 26.6 | 0.066 | RIJC0216 | 6.49 | 26.8 | 0.047 |
| RIJC0069 | 3.90 | 28.5 | 0.039 | RIJC0217 | 3.90 | 22.6 | 0.013 |
| RIJC0070 | 4.60 | 27.8 | 0.062 | RIJC0219 | 6.78 | 27.9 | 0.094 |
| RIJC0071 | 3.97 | 25.8 | 0.161 | RIJC0220 | 3.91 | 28.2 | 0.049 |
| RIJC0072 | 4.73 | 29.7 | 0.046 | RIJC0223 | 3.91 | 24.8 | 0.150 |
| RIJC0073 | 3.90 | 28.0 | 0.084 | RIJC0224 | 3.90 | 26.7 | 0.033 |
| RIJC0074 | 3.90 | 23.5 | 0.122 | RIJC0225 | 5.77 | 24.7 | 0.074 |
| RIJC0076 | 4.69 | 28.1 | 0.039 | RIJC0228 | 5.77 | 24.3 | 0.050 |
| RIJC0078 | 3.90 | 22.0 | 0.053 | RIJC0229 | 3.92 | 28.1 | 0.043 |
| RIJC0079 | 5.76 | 27.1 | 0.053 | RIJC0230 | 4.58 | 28.9 | 0.033 |
| RIJC0080 | 3.91 | 21.7 | 0.076 | RIJC0231 | 5.91 | 24.4 | 0.088 |
| **Genotypes** | **PL-EM** | **EM-FL** | **PPSEN** | **Genotypes** | **PL-EM** | **EM-FL** | **PPSEN** |
| RIJC0232 | 4.79 | 33.1 | 0.088 | RIJC0320 | 3.90 | 30.1 | 0.097 |
| RIJC0233 | 4.79 | 23.9 | 0.100 | RIJC0322 | 4.85 | 23.0 | 0.107 |
| RIJC0234 | 6.38 | 24.3 | 0.125 | RIJC0324 | 5.67 | 29.1 | 0.035 |
| RIJC0235 | 4.99 | 30.5 | 0.058 | RIJC0326 | 5.75 | 25.4 | 0.048 |
| RIJC0236 | 5.66 | 28.1 | 0.066 | RIJC0327 | 4.79 | 22.8 | 0.130 |
| RIJC0237 | 4.79 | 27.4 | 0.059 | RIJC0328 | 6.49 | 28.3 | 0.060 |
| RIJC0238 | 3.90 | 27.6 | 0.040 | RIJC0330 | 5.43 | 25.6 | 0.056 |
| RIJC0239 | 5.35 | 23.7 | 0.091 | RIJC0333 | 5.74 | 21.9 | 0.128 |
| RIJC0243 | 4.79 | 28.3 | 0.069 | RIJC0334 | 4.79 | 31.3 | 0.063 |
| RIJC0245 | 4.78 | 22.5 | 0.101 | RIJC0335 | 4.79 | 22.1 | 0.093 |
| RIJC0246 | 5.86 | 24.3 | 0.099 | RIJC0337 | 3.90 | 28.4 | 0.041 |
| RIJC0247 | 3.90 | 27.5 | 0.088 | RIJC0339 | 5.76 | 31.7 | 0.045 |
| RIJC0248 | 4.79 | 23.0 | 0.179 | RIJC0340 | 6.38 | 25.6 | 0.116 |
| RIJC0249 | 4.79 | 29.7 | 0.052 | RIJC0342 | 5.83 | 27.0 | 0.039 |
| RIJC0250 | 4.18 | 23.1 | 0.137 | RIJC0343 | 7.36 | 26.3 | 0.059 |
| RIJC0251 | 4.80 | 23.4 | 0.056 | RIJC0344 | 4.03 | 30.4 | 0.061 |
| RIJC0252 | 5.75 | 24.1 | 0.149 | RIJC0346 | 3.91 | 28.0 | 0.084 |
| RIJC0253 | 5.87 | 24.7 | 0.106 | RIJC0347 | 4.73 | 34.5 | 0.055 |
| RIJC0254 | 3.91 | 23.1 | 0.118 | RIJC0348 | 4.75 | 24.1 | 0.139 |
| RIJC0255 | 3.91 | 26.0 | 0.105 | RIJC0349 | 3.92 | 30.3 | 0.073 |
| RIJC0257 | 4.57 | 21.4 | 0.094 | RIJC0350 | 4.79 | 28.7 | 0.054 |
| RIJC0259 | 5.75 | 28.3 | 0.084 | RIJC0352 | 4.03 | 27.5 | 0.041 |
| RIJC0260 | 5.34 | 23.9 | 0.127 | RIJC0353 | 4.60 | 27.7 | 0.050 |
| RIJC0261 | 5.76 | 26.9 | 0.123 | RIJC0355 | 4.79 | 27.4 | 0.082 |
| RIJC0262 | 5.76 | 32.6 | 0.067 | RIJC0356 | 3.90 | 28.0 | 0.068 |
| RIJC0264 | 4.14 | 29.7 | 0.087 | RIJC0357 | 4.93 | 31.7 | 0.028 |
| RIJC0301 | 3.91 | 28.7 | 0.094 | RIJC0358 | 5.72 | 31.8 | 0.100 |
| RIJC0302 | 5.75 | 25.9 | 0.081 | RIJC0360 | 4.79 | 29.2 | 0.097 |
| RIJC0303 | 4.62 | 30.1 | 0.042 | RIJC0362 | 4.73 | 29.1 | 0.050 |
| RIJC0306 | 3.90 | 26.9 | 0.072 | RIJC0363 | 4.79 | 23.3 | 0.145 |
| RIJC0311 | 4.79 | 23.8 | 0.134 | RIJC0364 | 5.74 | 21.3 | 0.114 |
| RIJC0313 | 3.90 | 24.8 | 0.104 | RIJC0366 | 3.91 | 24.3 | 0.020 |
| RIJC0314 | 4.79 | 26.5 | 0.131 | RIJC0367 | 3.90 | 23.2 | 0.081 |
| RIJC0316 | 3.91 | 24.0 | 0.045 | RIJC0368 | 4.79 | 23.2 | 0.105 |
| RIJC0317 | 5.68 | 29.4 | 0.059 | RIJC0370 | 4.79 | 30.1 | 0.035 |

| **Table S3. Summary of QTL mapping results.** GSPs related to time-to-flowering in the DSSAT Bean-CROPGRO models were mapped in the RIF. The GSPs included PL-EM (Planting to emergence), EM-FL (Emergence to flowering) and PPSEN (Photoperiod sensitivity). | | | | | | |
| --- | --- | --- | --- | --- | --- | --- |
| **GSP** | **Chrom.** | **Marker Closest to QTL** | **Map Position (cM)** | **LOD** | **Effect %** | **QTL Overlap*** |
| PL-EM | 2 | DiM2-46 | 96.6 | 3.38 | 7.4 | - |
| PL-EM | 3 | DiM3-47 | 85.9 | 3.82 | 7.6 | - |
| PL-EM | 7 | DiM7-2 | 6.7 | 6.54 | 14.5 | - |
| EM-FL | 1 | DiM1-11 | 20.7 | 16.50 | 28.5 | TF1 |
| EM-FL | 1 | Fin | 41.6 | 26.40 | 45.0 | TF2 |
| EM-FL | 3 | DiM3-27 | 48.4 | 9.10 | 4.7 | TF6 |
| EM-FL | 11 | DiM11-9 | 18.3 | 3.42 | 2.2 | - |
| PPSEN | 1 | DiM1-29 | 59.8 | 23.31 | 53.2 | TF3 |

*Indicates whether the QTL for a GSP overlaps with a “time-to-flowering” QTL reported earlier by Bhakta et al.**^17^**

| **Table S4. Allele operators of the TF QTLs.** These operators are used in the simulation of temperature and day length responses through the implementation of the fixed-effects model function described by Eq. [2]. The J-CTF3 and J-CTF1, 2, 3 correspond to Jamapa synthetic genotypes carrying Calima allele replacements at QTLs TF1 and TF1,2,3, respectively. | | | | | | | | | | | | |
| --- | --- | --- | --- | --- | --- | --- | --- | --- | --- | --- | --- | --- |
| **Genotype** | **TF1** | **TF2** | **TF3** | **TF4** | **TF5** | **TF6** | **TF7** | **TF8** | **TF9** | **TF10** | **TF11** | **TF12** |
| Calima | 1 | 1 | 1 | 1 | 1 | 1 | 1 | 1 | 1 | 1 | 1 | 1 |
| Jamapa | -1 | -1 | -1 | -1 | -1 | -1 | -1 | -1 | -1 | -1 | -1 | -1 |
| RIJC031 | -1 | -1 | -1 | -1 | 1 | 1 | 1 | -1 | -1 | 1 | 1 | 1 |
| RIJC366 | 1 | 1 | -1 | -1 | 1 | 1 | 1 | -1 | 1 | -1 | -1 | -1 |
| J-CTF3 | -1 | -1 | 1 | -1 | -1 | -1 | -1 | -1 | -1 | -1 | -1 | -1 |
| J-CTF1,2,3 | 1 | 1 | 1 | -1 | -1 | -1 | -1 | -1 | -1 | -1 | -1 | -1 |

**Flowering Rate Model- R code**

rm(list=ls())

options(scipen = 999)

options(digits = 15)

## loading R libraries

library(lmerTest)

library(ggplot2)

library(dplyr)

### Setup your directory location

#setwd()

setwd("")

##Read input data

we <- read.table('Weather_daily.txt',header=T)

r1 <- read.table('RIL_R1data_weatherDAPtoFF.txt',header=T)

#######...................................

## Part 1 - mixed effect model parameter estimation

#######...................................

#mean for centering

sradMean <-mean(r1$Srad)

dayMean<- mean(r1$Day)

tminMean<- mean(r1$Tmin)

tmaxMean<- mean(r1$Tmax)

#centering weather related variables

r1$Srad_c<-r1$Srad-sradMean

r1$Day_c<-r1$Day-dayMean

r1$Tmin_c<-r1$Tmin-tminMean

r1$Tmax_c<-r1$Tmax-tmaxMean

r1$R1rate <- 1/r1$R1 ### 1/TFi variable

###### Linear Mixed-Effect Model for floweing Rate 1/TF #############################################

modelr1rate = lmer((R1rate)~Tmax_c+Tmin_c+Day_c+Srad_c+

TF1+TF2+TF3+TF4+TF5+TF6+TF7+TF8+TF9+TF10+TF11+TF12+

TF1*TF2+

Srad_c*TF12+

Day_c*TF3 +

Day_c*TF7+

Day_c*TF12+

Tmax_c*TF5+

Tmin_c*TF2+

Tmin_c*TF3+

(1|RIL),data=r1)

summary(modelr1rate)

anova(modelr1rate)

##output fixed effect

write.table( summary(modelr1rate)$coefficients,"fixedeffect_coeff.txt" , quote=F,row.names = F,sep="\t")

#output random effect

ranef(modelr1rate)

## predicting the R1 using Environment and QTL info from SAME DATASET used for model building and parameter estimation

r1$predRate <-predict(modelr1rate,newdata=r1)

summary(lm((r1$R1rate)~r1$predRate))$adj.r.squared

R.sqRateTf<-summary(lm((r1$R1rate)~r1$predRate))$adj.r.squared

## plotting observedRate vs predictated Rate

b<-ggplot(r1,aes(R1rate,predRate,color=SITE)) +

scale_x_continuous(limits = c(0.005, 0.045)) +

scale_y_continuous(limits = c(0.005, 0.045)) +

geom_point(size=2.5)+theme_bw(base_size=16) +

geom_abline(slope=1, intercept=0)+labs(x="Rate of Flowering",y="Predicted Rate of Flowering",title="Centered - Rate - mixed-effect")

b<- b+ annotate("text", x = 0.015, y = 0.04, label = paste("adj.Rsqare=",round(R.sqRateTf,2),sep=""))

b

########################################################################################################

# PART 2 >>> DAILY Prediction using mixed effect model - with Random effects forced to be zero

######################################################################################################

########....................................................................

# PART 2a : START-- Estimating daily flowering rate for each RIL at each SITE

########....................................................................

R1 <- read.table('All_R1data_weatherDAFtoFF.txt',header=T) # this input data file has data on parents and RIL tested in 2016 Citra

##remove jamapa & calima data and Citra 3_16 data

r11 <- droplevels(R1[(R1$SITE != "CIT3_16" & R1$RIL != "Calima" & R1$RIL != "Jamapa"),])

predictionlist <- list()

predictionlist[[1]] = rbind(predictionlist,c("SITE","RIL","o_R1", 'DayCount','CounterR1','dailyRateR1','stepper')) # first list row in prediction table--will be joined at the end of loop

rowcount = 1

stepper = 1

for (s in c("ND","CIT","PAL","POP","PR")){

r2<-subset(r11,r11$SITE==s) # subsetting RIL data file

we1 <- subset(we,SITE==s) # subsetting weather data file

##looping over SITE,RIL,DAY

for (i in 1:nrow(r2)) {

SITE <- c(as.character(r2$SITE[i]))

rsite <- SITE

RIL <- c(as.character(r2$RIL[i]))

o_R1 <- c(r2$R1[i])

TF1 <- c(r2$TF1[i])

TF2 <- c(r2$TF2[i])

TF3 <- c(r2$TF3[i])

TF4<- c(r2$TF4[i])

TF5<- c(r2$TF5[i])

TF6 <- c(r2$TF6[i])

TF7 <- c(r2$TF7[i])

TF8 <- c(r2$TF8[i])

TF9 <- c(r2$TF9[i])

TF10 <- c(r2$TF10[i])

TF11 <- c(r2$TF11[i])

TF12 <- c(r2$TF12[i])

CounterR1 = 0

DayCount = 0

for (j in 1:nrow(we1)){

DayCount <- we1$DAP[j]

# Adjusting weather variables

Srad_c <- c(we1$Srad[j])-sradMean

Day_c <- c(we1$newDAYLhr[j])-dayMean

Tmax_c <- c(we1$Tmax[j])-tmaxMean

Tmin_c <- c(we1$Tmin[j])-tminMean

r1day <- as.data.frame(SITE,RIL,TF1,TF2,TF3,TF4,TF5,TF6,TF7,TF8,TF9,TF10,TF11,TF12,Srad_c,Tmin_c,Tmax_c,Day_c)

## Estimating daily flowering rate gain based on "modelr1rate" model

flowerNow<-predict(modelr1rate,re.form=NA,newdata=r1day) ## prediction with random effect as zero

#flowerNow<-predict(modelr1rate,newdata=r1day) ## prediction with random effect

CounterR1 = CounterR1 + flowerNow[[1]] ## counting cumulative flowering rate

dailyRateR1 = flowerNow[[1]]

rowcount = rowcount + 1

predictionlist[[rowcount]] <- c(SITE,RIL,o_R1,DayCount,CounterR1,dailyRateR1,stepper)

## exiting loop when rate reaches 1.02

if (CounterR1[[1]] >1.02){break}

}

stepper = stepper +1

print(c(SITE,stepper))

}

}

predictionRate <- as.matrix(do.call("rbind", predictionlist)) # converting prediciton list to matrix

colnames(predictionRate)<-predictionRate[1,] # fixing header row names

predictionRate<-predictionRate[-1,] # removing old header row

##writing daily rate prediciton output file

write.csv(predictionRate,"Daily_rate_prediction_outputRIJC_Centered_withRandomEffectsAsZero.csv",row.names=F,quote = F)

########........................................................................................................................

# PART 2b : START --- Estimating the day on which flowering rate is estimated to be 1.0 START - Centered data with Random Effects as Zero

########.......................................................................................................................

##Read input file - daily rate prediction output.csv file

predictionRate <- read.csv("Daily_rate_prediction_outputRIJC_Centered_withRandomEffectsAsZero.csv",header=T)

#Plotting rate of flowering prediction(daily) for each ril at each Site

ggplot(predictionRate,aes(DayCount,CounterR1,group=RIL))+

facet_wrap( ~ SITE,ncol=2)+

geom_line(size=0.5,color="dark gray")+

geom_hline(yintercept =1.00,size=0.5,color="black")+

theme_bw(base_size=16)+

labs(x="Days",y="Predicted Rate of Flowering",title="Centered_with_Random_Effect_as_zero")

##interpolating data to estimate Day count when Rate hits 1.0 -- USE FOR RIL

GuesstheDay <- NULL

for (i in unique(predictionRate$RIL)){

subdata <- subset(predictionRate,RIL ==i & CounterR1 >0.94)

for (j in unique(subdata$SITE)){

frame <- subset(subdata,SITE == j)

#loop to find day when rate is 1.0

if (identical(which(frame$CounterR1==1.0), integer(0)) == "TRUE" ){

subframe <-frame[c(max(which(frame$CounterR1<1)),min(which(frame$CounterR1>1))),] ##picking a value before 1.0 and after 1.0

mod <- lm(data=subframe,DayCount~CounterR1) ## linear model to identify numeric day when rate =1.0

Rate_R1_DayPrediction <- round((mod$coefficients[[1]]+mod$coefficients[[2]]),2)

} else {

Rate_R1_DayPrediction <- round(frame$DayCount[which(frame$CounterR1==1.0)],2) # if rate 1.0 is in actual output then using that instead of interpolating.

}

o_R1<- frame$o_R1[[1]]

SITE <- frame$SITE[[1]]

RIL <- frame$RIL[[1]]

GuesstheDay = rbind(GuesstheDay, data.frame(SITE,RIL,Rate_R1_DayPrediction))

}

}

GuesstheDay$id <- paste0(GuesstheDay$SITE,"_",GuesstheDay$RIL)

raw<-read.table('RIL_R1data_weatherDAPtoFF.txt',header=T)

raw$id <-paste0(raw$SITE,"_",raw$RIL)

GuesstheDay2<- merge(x = GuesstheDay, y = raw[ , c("id", "R1")], by = "id", all.x=TRUE)

colnames(GuesstheDay2) <- c("ID", "SITE", "RIL", "Predicted_Day_when_R1rate_is_1.0_REisZero","obs_R1")

### writing output to directory -- estimated day when the predicted flowering rate hit 1.0

write.table(GuesstheDay2,"r1_rate1.0_Prediction_center_RIL_Centered_withRandomEffect_as_Zero.txt",row.names=F,quote = F,sep='\t')

## plotting observedRate vs predictated Rate

b<-ggplot(GuesstheDay2,aes(obs_R1,Predicted_Day_when_R1rate_is_1.0_REisZero,color=SITE)) +

scale_x_continuous(limits = c(20, 80)) +

scale_y_continuous(limits = c(20, 80)) +

geom_point(size=2.5)+theme_bw(base_size=14) +

geom_abline(slope=1, intercept=0)+labs(x="Observed R1 Days",y="Predicted R1 Days_ratebased",title="Centered_with_Random_Effect_as_zero")

modrate<-lm(data=GuesstheDay2,obs_R1~Predicted_Day_when_R1rate_is_1.0_REisZero)

summary(modrate)$adj.r.squared

adj.r <- summary(modrate)$adj.r.squared

b<- b+ annotate("text", x= 30, y=70,label = paste("adj.Rsqare=",round(adj.r,2),sep=""))

b

########........................................................................................................................

# PART 2b : END --- Estimating the day on which flowering rate is estimated to be 1.0 START - Centered data with Random Effects as Zero

########.......................................................................................................................

INPUT DATA FILES

‘Weather_daily.csv’

‘R1data_weatherDAPtoFF.csv’

‘AllQTLcombo.csv’

**Rate of Flowering Sub-Module.** This sub-module computes the daily rate of progress from planting to the day when the first flower appears on the plants. The FORTRAN source code for the RF Module is listed below. The “!” on lines indicate that the line is a comment (for example, see line 2 below). This module is run once per day to compute the rate of development for the specific genotype being simulated in the day’s environmental conditions. Referring to line numbers in this code, Line 18 contains the environmental inputs into this module for each day, which are day length (DL_i_), solar radiation (Srad_i_), and maximum and minimum temperatures (Tmax_i_ and Tmin_i_). Another key input to the module is the vector (TF), which has the genetic information (QTL allele operators) for the RIL being simulated. This Line 18 also shows the output that is communicated back to the main driver, SumRF_ij_, which is computed by accumulating the daily values of RFij for the RIL, starting at the time of planting when SumRF_ij_ = 0.00. Lines 26 – 30 set this initial value on the first day of the simulation, assuming that the program starts on the day of planting. Lines 36 - 40 set the mean values of environmental variables across all five locations as experienced during each RIL’s developmental period between emergence and first flower appearance.

The equation that computes daily rate of development for the genotype and environmental conditions being considered is given in Lines 45 – 67. The equation shown in the **RF Module** was based on Eqs. [2] and [3]. This new dynamic version describes more of the variability in the data than did the original mixed-effects linear model from Bhakta et al. (2017). Further, this dynamic version is critical to its implementation in other environments where future weather is not known in real-time predictions and where environmental conditions differ from those in the multi-environment trials used to estimate the model. Also, note that one can replace the model shown here with another function that computes each day's rate of progress toward first flower, such as using the original bean rate of progress toward flowering model**^13^** or the dynamic gene-based model published by Wallach et al.**^31^** Finally, Lines 85 – 86 in the **RF Module** compute SumRF_ij_, the summation of the daily development rates for environment *i* and genotype *j*, the integration step given in Eq. [3] in the paper. The remaining lines in this module (92 – 140) define variables to help document the program; these lines are not executed when the bean model is run.

**Fortran Code for the Rate of Flowering Sub-Module.** Below is a listing of the FORTRAN computer code for computing daily rates of development after emergence up to the time of first flower.

! Written, 2017-01-05 by Jim Jones

! With assistance from CH Porter

! Based on linear mixed effects models developed by Mehul Bhakta (Equations [2] and [3].

! Module for Rate of Development toward first flower in Common Bean, using G, E, and G x E inputs

! Integrating RF vs. time to simulate first flower event

! Computes daily rate of development, accumulates it and passes

! the cumulative value back to the calling main program

! Inputs to this module are daily day length (DLi, h),

! daily maximum temperature (Tmaxi, C), daily minimum temperature (Tmini, C),

! daily mean solar radiation (Sradi, MJ/m2), QTLs (TFi), and prior cumulative development (SumRFi)

! Based on data and relationships from Vallejos NSF project, using data from 5 locations,

! Used M. Bhakta RF daily model fitted to RF = (1/TF) depending on E, G, and GxE factors

!------------------------------------------------------------------------

Subroutine RFlower_rate (DLi, Sradi, Tmaxi, Tmini, TF, SumRFij)

Real, intent(in) :: DLi, Sradi, Tmaxi, Tmini !daily weather data

Real, dimension(70) :: TF !Vector of cultivar parameters, genotype i

Real, intent(out) :: SumRFij !progression towards anthesis, env i, genotype j

Real RFij, RFmean, DLm, Sradm, Tmaxm, Tminm

logical first

data first /.true./

if (first) then

SumRFij = 0.0

first = .false.

endif

!Averages across 5 env in datasets used to estimate model

! Mean values of environmental variables, estimated during fitting process for specific dataset used

! Values from M. Bhakta based on integrated rate model. Received 8/19/2018

RFmean = 0.02351

DLm = 12.370

Sradm = 18.218

Tmaxm = 27.458

Tminm = 16.128

!------------------------------------------------------------------------

! The dynamic gene-based mixed effects linear model, Equation [2]

!------------------------------------------------------------------------

!

RFij = RFmean

& + 5.966e-04 * (Tmaxi-Tmaxm)

& + 5.229e-04 * (Tmini - Tminm)

& - 1.542e-03 * (DLi - DLm)

& - 8.531e-05 * (Sradi - Sradm)

& + 9.383e-04 * TF(1)

& + 1.273e-04 * TF(2)

& - 6.810e-04 * TF(3)

& + 2.547e-04 * TF(4)

& - 2.944e-05 * TF(5)

& + 5.599e-04 * TF(6)

& - 4.332e-04 * TF(7)

& - 2.159e-04 * TF(8)

& - 4.693e-04 * TF(9)

& - 2.746e-04 * TF(10)

& + 3.463e-04 * TF(11)

& - 1.723e-04 * TF(12)

& + 2.795e-04 * TF(1) * TF(2)

& - 2.749e-05 * (Sradi - Sradm) * TF(12)

& - 7.637e-04 * (DLi - DLm) * TF(3)

& - 1.527e-04 * (DLi - DLm) * TF(7)

& - 1.401e-04 * (DLi - DLm) * TF(12)

& + 9.801e-05 * (Tmaxi-Tmaxm) * TF(5)

& - 8.183e-05 * (Tmini - Tminm) * TF(3)

& - 2.981e-06 * (Tmini - Tminm) * TF(2)

!

! Note that one can replace the above mixed effects linear model with

! any function that computes each day's rate of progress toward 1st flower

! including use of the original bean rate of progress toward flowering model

!------------------------------------------------------------------------

! Compute time integral of development to pass back as cumulative

! progress toward development each day

! In the equation for computing SumRFij, the time step is assumed

! to be 1.0 d for this module (fixed)

!------------------------------------------------------------------------

! Limit rate of development to positive values;

! initial value=0.0. When SumRFij first reaches 1.00,

! flowering will occur

if (RFij < 1E-5) RFij = 0.0

SumRFij = SumRFij + RFij*1.0

Return

End Subroutine RFlower_rate

!------------------------------------------------------------------------

! Variable Definitions:

!------------------------------------------------------------------------

!

! RFmean = 0.02342 is the mean rate toward flowering (1/day) across the 5 site-years in the bean dataset

! DLi = day length during time from sowing to flowering observed for the ith genotype (hours),

! DLm = mean day length across all five sites, all genotypes (12.37 hrs),

! Sradi = average solar radiation from sowing to flowering observed by the ith environment (Srad, MJ/m2d),

! Sradm = mean solar radiation across all sites, genotypes and transplanting dates in these data (18.218 MJ/m2d),

! Tmaxi = daily maximum temperature each day during time from planting to first flower

! Tmaxm = average of Tmaxi from transplanting to first flower across all genotypes, sites, years, (27.458 °C),

! Tmini = daily minimum temperature each day during time from planting to first flower

! Tminm = mean of the daily minimum temperatures means across all treatments, from planting to 1st flower (16.128)

! TF(1):TF(70) = alleles at QTL TF1 :TF70 in jth genotype

!------------------------------------------------------------------------

! INPUTS to Module:

!------------------------------------------------------------------------

! E(Daily Environmental variables): DLi, Sradi, Tmaxi, Tmini

! G (Genetic Variables): TF(1) thru TF(70); only 12 are

! used in the linear model developed by Bhakta et al. for Bean

! The numerical coefficients in the equation were developed for this

! specific bi-parental population. These are either coded in the equation

! above as constant numerical values

! that cannot be changed by users for other genotypes

! Changing them would cause results outside the confines of the data

! used to estimate them. One can change TFj to compute TF for a particular line

! Initial value of progress toward development

! SumRFij0 (initially, SumRFij0 = 0.0 at time of transplanting)

!------------------------------------------------------------------------

! OUTPUTS from Module, Dynamic Variables:

!------------------------------------------------------------------------

! SumRFij = current progress toward first flowering from emergence

! (dimensionless), integral of RFij from crop emergence or

! planting to current day (environment i, genotype j)

! The starting time for accumulation of time to first flower depends

! on the data used to fit the model. Ideally, it will be emergence.

!------------------------------------------------------------------------

! Local variables:

!------------------------------------------------------------------------

! RFij = Daily rate toward development, fraction such that when integrated over time and a value of 1.0 is reached (or exceeded

! First Flower occurs on that day, this is the mixed effects dependent variable

! RFij = Daily rate of progress from planting to first flower appearance for selected

! genotype (j) and environmental factors on the current environment & day (i)

!------------------------------------------------------------------------

**Driver Sub-Module.** This sub-module controls the sequence of calculations as it runs the *Rate of Flowering Sub-Module.* Thus, it implements the dynamic bean rate of flowering model and demonstrates how the model uses the MET data to predict time to first flower. In this implementation, the **Driver Sub-Module** was designed to conduct a simple sensitivity analysis to study specific genotypic responses to varying day lengths and daily temperatures. This module produces output files that have results of time to first flower for each specified environmental condition for a given genotype. One can use this module to identify the allelic combination(s) that will determine a specific time to first flower under predefined environmental conditions, including those predicted by climate warming models. These output files can be used to produce graphs of time to first flower responses as a function of temperature and/or day length conditions.

The module first sets the genetic values for the RIL to be simulated (Lines 33 – 44). It then opens a file into which model outputs are to be written and writes text headers for variables that will be written to the files (Lines 52 – 72). Lines 74 – 83 set initial conditions for a new combination of G and E. Note that one could also read daily values from a data file in order to simulate time to flower under time -varying weather conditions (Lines 46 – 51). Lines 88 - 100 is where the daily weather conditions are specified for each day of each intended combination of G and E for the simulation. Because this program is designed to simulate time to first flower over a range of environments, Lines 108 – 129 contain loops to set values of day length (Lines 108 – 112) and temperature (Lines 114 – 122). For each combination of day length and temperature, the model simulates time of flowering for the selected genotype. Line 138 calls the RF module to run the model. Line 143 determines when the SumRF_ij_ first exceeds 1.0 for a given set of G and E conditions, and this day is the day when the first flower occurs. Lines 145 – 152 write outputs for the simulated conditions. This program loops back through the driver module to simulate the selected combinations of day length and temperature. To simulate a different RIL, the QTLs for the specific genotype must be specified in Lines 33 – 44. There are 12 QTL allele operators to set, which a value of +1 for each QTL representing the Calima parent and a value of -1 for the Jamapa parent. Other RILs have different combinations of +1 and -1 numerical values.

**Driver Sub-Module Code.** The driver main flowering sub-module contains the FORTRAN code listing developed to simulate dynamic time to first flower model using daily weather value inputs of day length, maximum and minimum temperature, and solar radiation. Comment lines have an “!” at the front of the line or in front of the comment on a line.

!******************************************************************************

!* Driver Module for running the RF Module to predict time of flowering *

!* Written, 2016-09-27 CH Porter, Jim Jones *

!******************************************************************************

Program Main

!------------------------------------------------------------------------

implicit none

integer DOY, SDat, YR, Fdoy, RunNo

integer Tmaxi_N, Tmini_N, DLi_N, k, m, i

real DLi, Sradi, Tmaxi, Tmini, ADAP

real DLi_incr, DLi_start

real Tmaxi_incr, Tmaxi_start

real Tmini_incr, Tmini_start

real SumRFij

real, dimension(70) :: TF !Vector of genotype markers / parameters

character(8) CultivarID

character(12) Weather

character(1) text

!------------------------------------------------------------------------

! Sensitivity tests using each marker to be +1 (Calima) or to be -1 (Jamapa)

!------------------------------------------------------------------------

CultivarID = "C=T1,2,3" !Jamapa values are all = -1, Calima = +1

TF = 0.0

! TF1 TF2 TF3 TF4 TF5 TF6 TF7 TF8 TF9 TF10 TF11 TF12

! 1 1 1 1 1 1 1 1 1 1 1 1 !Calima

! -1 -1 -1 -1 1 1 1 -1 -1 1 1 1 !RIJC031

! 1 1 -1 -1 1 1 1 -1 1 -1 -1 -1 !RIJC366

! -1 -1 -1 -1 -1 -1 -1 -1 -1 -1 -1 -1 !Jamapa

TF(1) = 1.0

TF(2) = 1.0

TF(3) = 1.0

TF(4) = -1.0

TF(5) = -1.0

TF(6) = -1.0

TF(7) = -1.0

TF(8) = -1.0

TF(9) = -1.0

TF(10)= -1.0

TF(11)= -1.0

TF(12)= -1.0

!------------------------------------------------------------------------

! Weather = "CCPO1201.WTH"

! initialization, Open Files for output

! NOTE: Could read daily weather data file here and replace constant env variables

! with daily-changing values

!------------------------------------------------------------------------

open(40,File='Sens.Temp.OUT',status='REPLACE')

write(40,'(A,A,17(/,2X,A,F8.3))') &

"Marker values for genotype: ", CultivarID, &

"TF(1) =", TF(1) , &

"TF(2) =", TF(2) , &

"TF(3) =", TF(3), &

"TF(4) =", TF(4), &

"TF(5) =", TF(5), &

"TF(6) =", TF(6), &

"TF(7) =", TF(7), &

"TF(8) =", TF(8), &

"TF(9) =", TF(9), &

"TF(10) =", TF(10), &

"TF(11) =", TF(11), &

"TF(12) =", TF(12)

write(40,'(/,a)') " Run# Cultivar DAYL Srad Tmax Tmin SumRFi ADAP ADOY"

write(*,'(/,a)') " Run# Cultivar DAYL Srad Tmax Tmin SumRFi ADAP ADOY"

!------------------------------------------------------------------------

! Set sowing/start day of year for flowering model to start

! Initialize progress toward flowering, SumDRi, & Day of First Flower, Fdoy

!------------------------------------------------------------------------

SDat = 90 !sowing date

ADAP = 0.0 !counter for number of days from sowing to anthesis

SumRFij = 0.0

Fdoy = 0

RunNo = 0

YR = 00

!------------------------------------------------------------------------

!Set constant values for weather inputs for sensitivity analysis

!------------------------------------------------------------------------

! doesn't iterate Sradi (uses mean values); currently, only Tmaxi, Tmini and DLi

Sradi = 18.218

Tmaxi_start = 14.0

Tmaxi_incr = 1.0

Tmaxi_N = 15

Tmini_start = 6.0

Tmini_incr = 1.0

Tmini_N = 15

DLi_start = 11.0

DLi_incr = 0.5

DLi_N = 15

open(30,File="Anthesis.OUT",status='REPLACE')

write(30,'(a)') "adap yr doy Srad Tmax Tmin SumRFij"

! write(*,'(a)') "adap yr doy Srad Tmax Tmin SumRFij"

!------------------------------------------------------------------------

!Daylength (DLi) variations loop, m counter, and set

!Temperature variations simulation loop, k counter

!------------------------------------------------------------------------

DLi = DLi_start

do m = 1, DLi_N !Daylength loop

! Increment daylength

DLi = DLi + DLi_incr

Tmaxi = Tmaxi_start

Tmini = Tmini_start

!------------------------------------------------------------------------

do k = 1, Tmaxi_N !Temperature loop

! Increment temperature

Tmaxi = Tmaxi + Tmaxi_incr

Tmini = Tmini + Tmini_incr

!------------------------------------------------------------------------

! Initialize accumulators

SumRFij = 0.0

ADAP = 0.0

RunNo = RunNo + 1

!------------------------------------------------------------------------

! daily time loop, each combination of T, DL

do i = 1,200

DOY = i !starting day of weather

if (DOY > SDat-1.and.SumRFij < 1.0)Then !Accumulate development after planting date (Sdat)

ADAP = ADAP+1

!------------------------------------------------------------------------

! call flowering module to calculate & integrate daily development rate over time

call RFlower_rate (DLi, Sradi, Tmaxi, Tmini, TF, SumRFij)

!------------------------------------------------------------------------

write(30,'(f5.1,1X,i2,1X,I3.3,3f6.2,f8.4)') ADAP, YR, DOY, Sradi, Tmaxi, Tmini, SumRFij

if (SumRFij > 1.0 .and. Fdoy < 1) then !First flower occurs

Fdoy = DOY

write(40,'(i6,2x,A8, 3f10.1,F10.2,2X,F10.5,F10.1,I10)') RunNo,CultivarID,DLi,Sradi,Tmaxi,Tmini,SumRFij,ADAP,Fdoy

write(*,'(i6,2x,A8, 3f10.1,F10.2,2X,F10.5,F10.1,I10)') RunNo,CultivarID,DLi,Sradi,Tmaxi,Tmini,SumRFij,ADAP,Fdoy

endif

endif

enddo !End daily time loop

!------------------------------------------------------------------------

ADAP = 0.0

SumRFij = 0.0

Fdoy = 0

enddo !end of Temperature loop

enddo !end of Daylength loop

!------------------------------------------------------------------------

Stop

End Program Main
